# *Plasmodium* actin-like proteins are essential for DNA segregation during male gametogenesis and malaria transmission

**DOI:** 10.1371/journal.ppat.1013687

**Published:** 2025-11-11

**Authors:** Aastha Varshney, Eisha Pandey, Nirdosh, Satish Mishra

**Affiliations:** 1 Division of Molecular Microbiology and Immunology, CSIR-Central Drug Research Institute, Lucknow, Uttar Pradesh, India; 2 Academy of Scientific and Innovative Research (AcSIR), Ghaziabad, Uttar Pradesh, India; Weill Cornell Medicine, UNITED STATES OF AMERICA

## Abstract

Protozoan parasites of the genus *Plasmodium* cause malaria and involve infection of multiple hosts and cell types during the life cycle. Producing sexually fit gametocytes is essential for transmitting the *Plasmodium* parasite into an anopheline mosquito vector. After the uptake of malaria parasites, male gametocytes undergo three rounds of DNA replication to produce eight nucleated flagellar gametes. Here, we report that the actin-like proteins Alp5a and Alp5b are involved in DNA segregation during male gametogenesis. The *Plasmodium*-specific Alp5a and Alp5b can be superimposed on human Arp2 and Arp3, localize to the nucleus, and interact with each other. Alp5a and Alp5b are individually dispensable for the development of *P. berghei* blood stages, but are simultaneously indispensable for parasite viability. Consistent with genetic studies, the inhibitory activity of the Arp2/3 complex inhibitor in *Plasmodium* supports an essential role for this complex during the blood stage. Deletion of Alp5a or Alp5b had no impact on actin nucleation, parasite growth, or gametocytemia during the blood stage. The knockout parasites were able to invade the mosquito midgut and form oocysts; however, these oocysts were significantly smaller in size and failed to mature, ultimately leading to their death. Genetic crosses revealed defects in male gamete integrity. We found that the reduced oocyst development was due to impaired DNA segregation during male gametogenesis. Our study provides molecular insights into the fundamental requirements of the Alps in *Plasmodium*, which are essential for malaria transmission.

## Author summary

The Arp2/3 complex, comprising seven subunits (ARPC1–5, Arp2, and Arp3), orchestrates actin polymerization by nucleating branched actin networks. This complex is pivotal for spindle actin assembly during mitosis and meiosis, thereby ensuring accurate chromosome segregation. Transmission of the malaria parasite relies on a highly synchronized and rapid process known as male gametogony. Gametocytes formed during the asexual blood stage are activated in the mosquito midgut, where male gametogenesis involves three rapid rounds of DNA replication and mitosis, resulting in an octoploid genome. However, the mechanisms ensuring equal DNA partitioning during this process remain poorly understood. In this study, we studied two actin-like proteins, ALP5a and ALP5b, which interact with each other and show structural similarity to human Arp3 and Arp2. We demonstrate that the Alp5 subunit of the Arp2/3 complex localizes to the nucleus and is essential for proper DNA segregation during male gametogenesis. These findings reveal a critical role for the Alp5 proteins in nuclear division during *Plasmodium* male gametogenesis and identify them as a potential target for transmission-blocking interventions against malaria.

## Introduction

Malaria parasites have a complex life cycle involving asexual and sexual replication in vertebrate and mosquito hosts. The sexual phase (gametogony) begins in the blood of the vertebrate host, whereas gametogenesis and meiosis require transmission to the mosquito host [1,2]. There are four periods of mitotic DNA synthesis and a single meiotic stage during the *Plasmodium* life cycle [1]. In the blood stage, parasites proliferate via a process called schizogony. Closed mitosis during schizogony involves repeated rounds of nuclear division to form a multinucleated cell (schizont), followed by a single round of cellularization that produces numerous merozoites [3–5]. The first cellular feature of schizogony is the formation of the hemispindle. The hemispindle is an array of microtubules that radiate into the nucleus from a single microtubule organizing center (MTOC) embedded in the nuclear membrane [6,7]. Once microtubules are formed, the spindle must assemble bipolarly to segregate the replicated chromosomes. The hemispindle retracts, and the MTOC duplicates to form a full, bidirectional mitotic spindle [8]. Mitosis is regulated by cyclin-dependent kinases (CDKs), NIMA (Never In Mitosis)-related (NEK), and Aurora, CDK-related kinases [9–11].

The transmission of malaria parasites to their mosquito vectors relies upon the parasite switching from asexual reproduction to sexual reproduction [12]. Male and female gametocytes produced during asexual schizogony are activated to begin gametogenesis to produce male and female gametes in the mosquito midgut [11]. After activation, microgametocytes undergo three rounds of rapidly closed mitosis, increasing the DNA content from 1 N to 8 N. Concomitant spindle formation and chromosome segregation occur over 8–12 min without nuclear division, followed by karyokinesis and cytokinesis to bud eight microgametes [11,13]. Male gametogenesis is a complex process that is coordinated by well-established components such as mitogen-activated PK2 (MAPK2), serine-arginine PK1 (SRPK1) and calcium-dependent PK4 (CDPK4), cell-division cycle protein 20 (CDC20), as well as the metallo-dependent PP, PPM1 in *P. berghei* [11,14–16]. Other players include radial spoke protein 9 (RSP9) and Pb22, APC3 in *P. berghei* [5,17,18], p25α in *P. yoelii* [19] and the patatin-like phospholipase, PLP2 in *P. falciparum* [20]. During male gametogenesis, rapid rounds of mitosis are driven by the formation of microtubular spindles to accurately segregate chromosomes. The kinetochore marker, NDC80, is located at distinct foci in the nucleus, and extends to form a bridge across the entire nuclear body during DNA segregation [21]. The list of essential regulators of gametogenesis is reviewed in [11].

Actins are filament-forming, highly-conserved proteins essential for parasite development, host cell invasion, and male gametogenesis [22,23]. Malaria parasites express two actin isoforms, ubiquitous actin-1 and specialized actin-2 [24,25]. Actin-1 is an essential factor for *P. berghei* asexual blood stage development, and it cannot be deleted [26]. Actin-2 is dispensable in the blood stage but is essential for male gametogenesis [27]. In mutant parasites, male gametocyte DNA replicates normally, and axonemes assemble, but egress is severely blocked. This resulted in impaired exflagellation, and only a very small number of ookinetes were occasionally formed, but oocysts were not detected [27]. Swapping of the actin-2 open reading frame (ORF) with actin-1 resulted in partial complementation of the defects in male gametogenesis. The complemented parasites produced ookinetes and were able to form oocysts, but these oocysts remained small, and their DNA was undetectable on day 8 post-blood meal. It does not develop into sporogonic oocysts [25]. A report revealed that the F-actin isoform actin-2 forms within a few minutes after gametocyte activation and persists until the zygote transforms into an ookinete [28]. Actin-2 is associated with the nucleus both in the male gametocyte and the zygote and readily forms long filaments necessary for male gametogenesis. Actin-2 plays a specific, essential role in the maturation of microgametes and it polymerizes via the classical nucleation-elongation mechanism [23]. While earlier studies proposed that *Toxoplasma gondii* actin polymerizes via an isodesmic mechanism [29], recent research has demonstrated that TgAct1 forms long filaments characterized by rapid treadmilling [30]. However, actin polymerization in *Plasmodium* follows a nucleation-elongation mechanism rather than an isodesmic one [23,31,32]. Actin-binding proteins (ABPs) are known to control critical cellular processes. Key regulatory proteins include actin depolymerizing factors (ADFs) [33–35], which promote rapid actin turnover by binding to monomeric actin, formins [36], which nucleate actin filament assembly, and actin capping proteins (CPs) [37,38], which regulate filament length and dynamics. Other important regulators identified include Profilin [39], which is involved in sporozoite motility and invasion. The actin nucleation factor Arp2/3 complex is characterized in other organisms. Malaria parasites have a minimal set of proteins that potentially regulate microfilament dynamics and lack canonical actin-nucleating factors such as the Arp2/3 complexes [40]. The limited sequence conservation of *Plasmodium* proteins is one of the reasons for knowledge gaps [41]. In fact, many proteins are currently considered absent or referred to as uncharacterized proteins in *Plasmodium*, such as the actin nucleator actin-related protein 2/3 (Arp2/3) complex [42–45].

The Arp2/3 complex nucleates actin filaments and comprises seven proteins with five unique polypeptides, called ARPC1–5, supporting a dimer of two Arps (actin-related proteins Arp2 and Arp3) [42,43,46]. The complex is conserved throughout the eukaryotic kingdom. However, its function has been validated in a few model organisms [47]. The Arp2/3 complex plays a role in actin polymerization, coordinating the nucleation of spindle actin during mitosis and meiosis and accurate chromosome segregation [48–51]. The Arp2/3 complex itself has been conserved throughout evolution [52,53], and it was assumed that the complex has been lost in apicomplexan parasites except ARPC1/ARC40 in malaria parasites [44,45]. A recent study identified a non-canonical Arp2/3 complex in *Plasmodium* composed of only five subunits, which localizes to endomitotic spindles and interacts with kinetochores [40]. They identified ARPC1 and Alp5b as components of this non-canonical Arp2/3 complex, essential for DNA segregation [40]. Our study independently confirms that Alp5a and Alp5b are related to human Arp3 and Arp2, respectively. We further demonstrate that Alp5b is critical for DNA segregation during male gametogenesis and expand upon these findings by disrupting Alp5a, revealing that it plays an equally essential role. Additionally, we found that Alp5a and Alp5b are individually dispensable during the blood stage but not when both are deleted simultaneously. While Alp5 knockout parasites can form ookinetes, their oocysts show a delayed death phenotype that ultimately blocks malaria transmission. Genetic crosses identified the underlying defect as male gamete impairment, specifically due to defective DNA segregation during male gametogenesis.

## Results

### *Plasmodium* Alp5a and Alp5b are structurally similar to human Arp2 and Arp3, localize to the nucleus, and interact with each other

**Fig 1:**
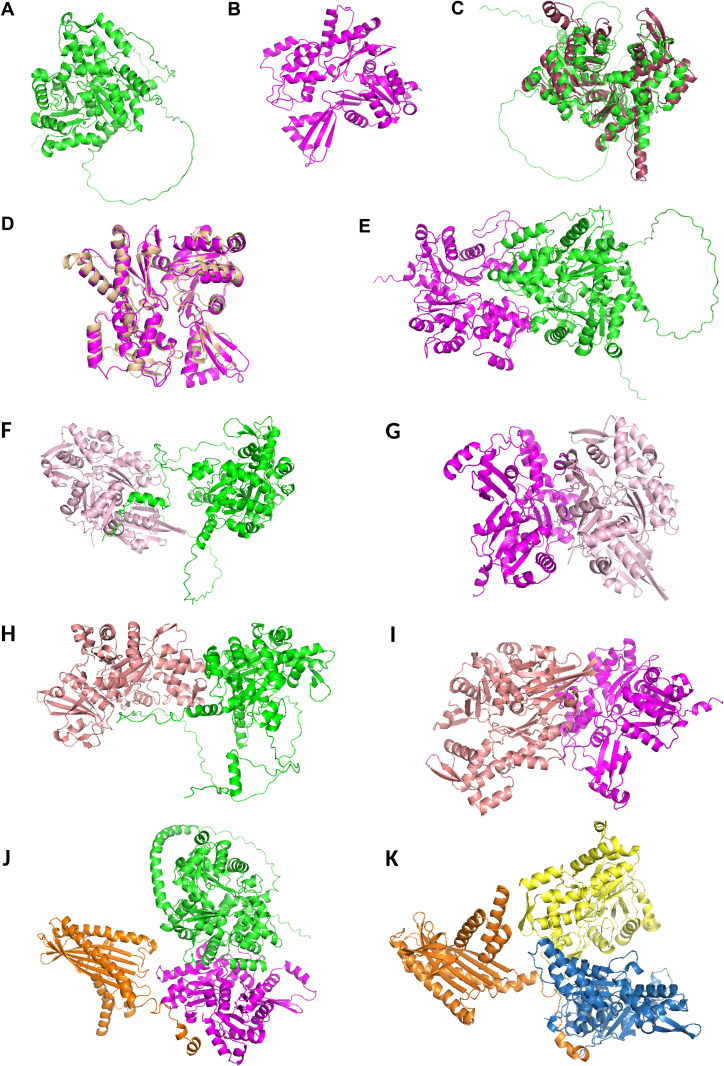
*Plasmodium* Alp5s are structurally conserved actin-related proteins (Arps). **(A)** The cartoon structure of *P. berghei* Alp5a was visualized using PyMOL, with α-helices, β-sheets, and loop regions depicted to highlight the protein’s overall fold and key secondary structural features. **(B)** Cartoon structure of Alp5b. **(C)** The structural superposition of *P. berghei* Alp5a (green) with human Arp3 (chocolate) reveals a RMSD of 2.961 Å. This RMSD value quantifies the degree of structural similarity between the two proteins, indicating both conserved regions and conformational differences. **(D)** Structural superposition of *P. berghei* Alp5b (magenta) and human Arp2 (brown) reveals a RMSD of 1.328 Å, indicating a high degree of structural conservation. This similarity suggests that key functional domains are maintained across species, despite evolutionary divergence. **(E)** The interaction between *P. berghei* Alp5a and 5b, as predicted by ClusPro. The docking model illustrates the predicted binding interface and interaction sites between the two proteins, highlighting their potential functional relationships. **(F)** Predicted interaction between *P. falciparum* Actin I (*Pf*Actin I; PDB ID: 6I4H, pink) and *P. berghei* Alp5a (green). **(G)** Interaction between PfActin I (6I4H; pink) and PbAlp5b (magenta). **(H)** Modeled complex of PbActin II (*Pb*Actin II; PDB ID: 6I4M, salmon red) with *Pb*Alp5a (green). **(I)** Predicted interaction between *Pb*Actin II (6I4M, salmon red) and *Pb*Alp5b (magenta). **(J)** Predicted ternary complex of PbAlp5a (green) and PbAlp5b (magenta) interacting with F-actin (orange), illustrating their potential binding interfaces and relative orientations. **(K)** Predicted ternary complex of Arp2 (yellow) and Arp3 (sky blue) bound to F-actin (orange), representing a canonical actin-branching complex.

We started our study by in silico analysis of the paralogous genes Alp5a (PBANKA_0811800) and Alp5b (PBANKA_1007500). NCBI BLASTP analysis of *P. berghei* Alp5s revealed that Alp5a and Alp5b are closely associated with *P. yoelii* and *P. falciparum,* respectively (S1 Fig). The protein-protein interactions of both Alp5a and Alp5b were confirmed via the STRING database. An interactome of 11 proteins revealed that all the protein interactions were the same for Alp5a and Alp5b, except for PBANKA_1434600 (formin2, putative protein) in Alp5a (S2 Fig). All interacting proteins were classified as conventional or actin-like proteins, dynactin subunits, formins, or subunits of the Arp2/3 complex. Notably, the presence of capping protein subunits in the interactome further highlights the diversity of actin-associated regulators identified (S2 Fig). The predicted protein structures of Alp5a and Alp5b were generated using AlphaFold2 and evaluated with UCLA-SAVES. The highest-quality models were subsequently selected for further analysis. (Fig 1A and 1B). Alp5a and Alp5b were superposed with the human Arp3 and Arp2 structures via PyMOL. We observed the superposition of Alp5a and Alp5b with human Arp3 and Arp2, with RMSD values of 2.961 Å and 1.328 Å, respectively (Fig 1C and 1D). The alignment at residue Phe425 of ALP5a (atom no. 2249) and residue 282 of Arp3 (atom no. 6856) highlights a key region with an RMSD of 2.961 Å, indicating potential functional relevance (S3 Fig). To investigate the potential interaction between Alp5a-Alp5b and Alp5-actin, protein–protein docking was performed using the ClusPro platform, and the resulting complexes were visualized using PyMOL. Interaction scores between Alp5a and Alp5b suggest a potential protein–protein interaction (Fig 1E). To assess the interaction of Alp5 with actin, we generated a 3D model of actin isoforms (actin 1 and actin 2) (S4A and S4D Fig). Cluspro protein-protein docking studies indicated that Alp5a has a stronger affinity for both actin isoforms than Alp5b (S4B, S4C, S4E and S4F Fig). To further validate these findings, we conducted additional computational analyses using experimentally determined high-resolution crystal structures of *P. falciparum* actin 1 (PDB ID: 6I4H; 1.40 Å resolution) and *P. berghei* actin 2 (PDB ID: 6I4M; 1.87 Å resolution), obtained from the RCSB Protein Data Bank. To assess the accuracy of AlphaFold2-predicted models relative to these experimental structures, structural alignments and superpositions were carried out. Root-mean-square deviation (RMSD) values were calculated to quantify structural similarity. The AlphaFold2-predicted structure of *P. berghei* actin 1 exhibited strong concordance with the *P. falciparum* actin 1 crystal structure (6I4H), with an RMSD of 0.627 Å. Similarly, the AlphaFold2 model of *P. berghei* actin 2 closely aligned with its corresponding crystal structure (6I4M), with an RMSD of 0.796 Å (S5 Fig). These low RMSD values indicate a high degree of structural similarity and support the reliability of the AlphaFold2 predictions. Further docking studies using experimentally resolved actin structures revealed interaction patterns and docking scores comparable to those observed with AlphaFold2-predicted models (Fig 1F–1I). Subsequent docking of the Alp5a–Alp5b complex with F-actin suggested that the C-terminus of Alp5b may mediate the interaction with F-actin. In parallel, docking analyses with the Arp2/3 complex indicated that F-actin primarily interacts with the Arp3 subunit (Fig 1J–1K). To assess overall structural similarity, the Alp5a–Alp5b complex was individually aligned with Arp2/3 complex subunits. Structural superposition of Alp5a with Arp3 yielded a root-mean-square deviation (RMSD) of 2.905 Å, whereas alignment of Alp5b with Arp2 produced an RMSD of 1.899 Å (S6 Fig). These relatively low RMSD values indicate a notable degree of structural similarity, supporting the notion that the Alp5a–Alp5b complex may adopt a conformation resembling that of the Arp2/3 complex. The AlphaFold3 predicted template modeling (pTM) score and interface predicted template modeling (ipTM) score are summarized in S2 Table.

**Fig 2:**
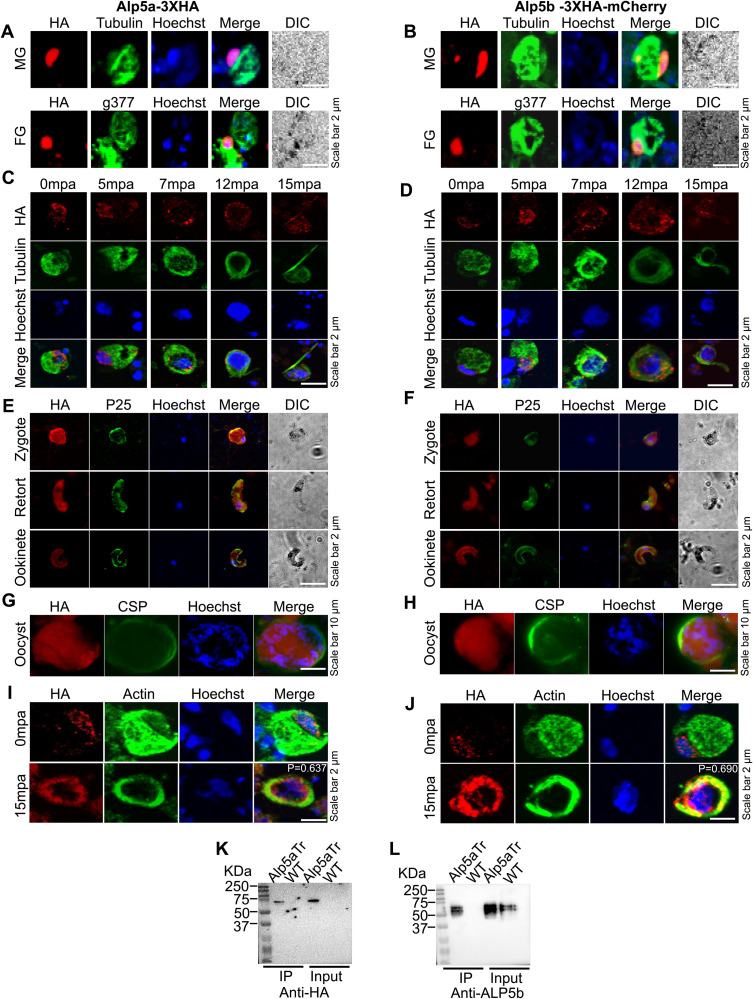
Expression and localization of Alp5s and their interaction. **(A and B)** Representative confocal immunofluorescence images of male and female gametocytes immunostained with anti-HA, anti-tubulin and anti-g377 antibodies. The Alp5a-3XHA and Alp5b-3XHA-mCherry parasites show their localization. Nuclei were stained with Hoechst. DIC: differential interference contrast. **(C and D)** Confocal immunofluorescence analyses of microgametes after gametocyte activation. The cells were fixed and immunostained with anti-HA and anti-tubulin antibodies. Alp5a and Alp5b localize from the nucleus to a microtubule-rich structure, likely the spindle or axoneme. **(E and F)** Immunofluorescence analyses of Alp5a-3XHA and Alp5b-3XHA-mCherry revealed their localization at the zygote, retort, and ookinete stages. Parasites were immunostained with anti-HA and anti-P25 antibodies. **(G and H)** Expression of Alp5a-3XHA and Alp5b-3XHA-mCherry parasites in oocysts. The midgut oocysts were labeled with anti-HA and anti-CSP antibodies. **(I and J)** Male gametocytes of Alp5 transgenic parasites were immunostained with anti-HA and anti-actin antibodies at 0- and 15-min post-activation, and subsequently imaged using a confocal microscope. At 0 minutes post-activation (mpa), Alp5 was predominantly localized to the nucleus, whereas actin was distributed in both the cytosol and the nucleus. Following activation at 15 mpa, Alp5 and actin are redistributed around the nucleus and exhibit colocalization. **(K and L)** Interaction of Alp5s in the parasite. The protein complex was immunoprecipitated from gametocytes of Alp5a-3XHA transgenic parasites using anti-HA magnetic beads. The Alp5a and Alp5b bands were detected by immunoblotting with anti-HA and anti-Alp5b antibodies.

Next, to check the expression, localization, and interactions of Alp5, we endogenously tagged Alp5a and Alp5b with 3XHA and 3XHA-mCherry, respectively (S7A, S7B, S7D, and S7E Fig). Western blot analysis with an anti-HA antibody confirmed the expression of correctly sized Alp5 fusion proteins in the gametocytes of the transgenic parasites (S7C and S7F Fig). IFA revealed the expression of Alp5a and Alp5b in male and female gametocytes but not in the ring or trophozoite stages. Male and female gametocytes were immunostained with anti-tubulin and anti-g377 antibodies to confirm gamete-specific expression. Both Alp5a and Alp5b were expressed predominantly in the nuclei of gametocytes (Fig 2A and 2B). The signals of Alp5a and Alp5b localize from the nucleus to a microtubule-rich structure, likely the spindle or axoneme (Fig 2C and 2D). Diffuse signals were also observed in the parasite cytoplasm. The expression of Alp5a and Alp5b was also detected in zygotes, ookinetes, and oocysts (Fig 2E-2H). The expression of Alp5a and Alp5b was not detected in sporozoites or exo-erythrocytic forms (S8A and S8B Fig). To assess the colocalization of Alp5 and actin, nonactivated and activated gametocytes were immunostained with anti-HA and anti-actin antibodies. The colocalization of Alp5 and actin in activated gametocytes indicated their possible interaction (Fig 2I and 2J). To determine the endogenous interaction between Alp5a and Alp5b, the complex was immunoprecipitated from gametocytes of Alp5a-3XHA transgenic parasites using anti-HA magnetic beads. The blot was probed with an anti-HA antibody that detected the Alp5a band (Fig 2K). The blot was reprobed with an anti-Alp5b antibody, which detected the Alp5b band, indicating the interactions of Alp5a with Alp5b (Fig 2L). These results suggest that Alp5 is predominantly expressed in the sexual stages and that the Alp5a and Alp5b subunits interact with each other.

### Alp5a and Alp5b are functionally redundant, and the Arp2/3 complex formed by these proteins is possibly essential for parasite survival‌

**Fig 3:**
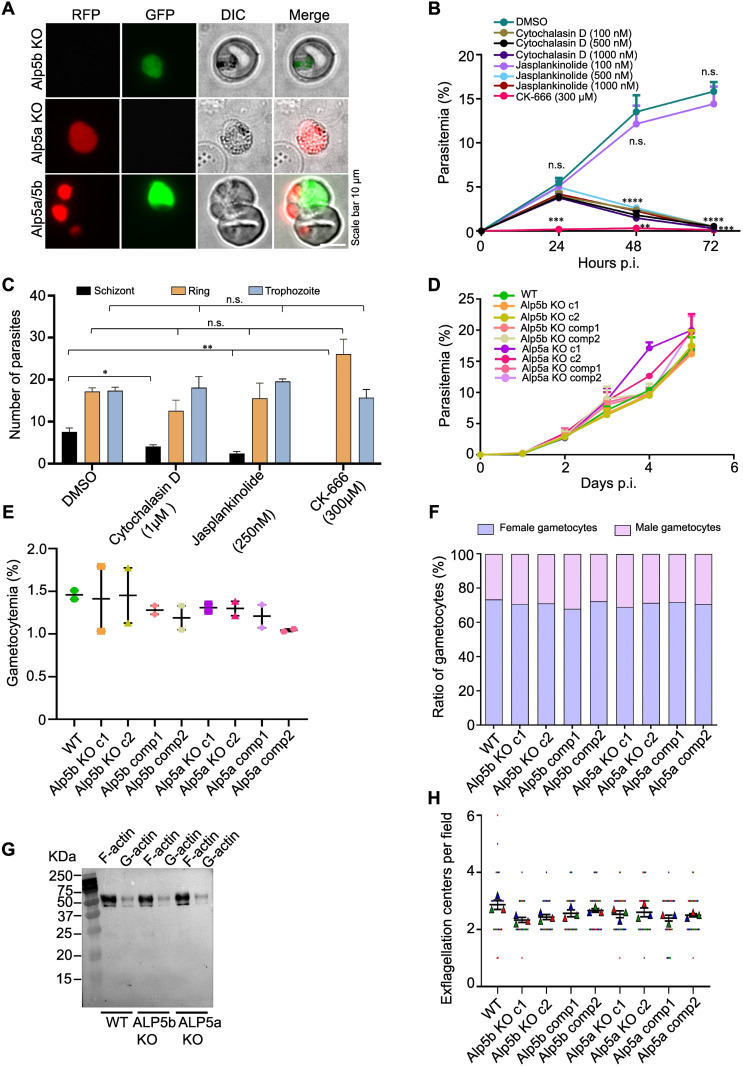
Alp5a and Alp5b are not individually required for asexual blood stage propagation, but simultaneously, they are indispensable for parasite viability. **(A)** Parasites transfected with constructs targeting Alp5a and Alp5b, tagged with mCherry and GFP respectively, predominantly gave rise to single knockout populations expressing only one of the two fluorophores. Rare instances of parasites expressing both mCherry and GFP were observed within the same red blood cell; however, the lack of fluorescence colocalization suggests that these are not true double knockouts. Representative images were acquired using a widefield fluorescence microscope. **(B)** Effect of inhibitors on the growth of *P. falciparum.* Parasitemia was measured at 24, 48, and 72 h after treatment with the indicated concentrations of cytochalasin D, jasplakinolide, and CK-666. At 24 h post-treatment, no significant difference in parasitemia was observed between DMSO-treated controls and cultures treated with either cytochalasin D or jasplakinolide (P = 0.2631; one-way ANOVA). Inhibition was observed in the CK-666-treated cultures, DMSO vs CK-666, ***P = 0.0008; unpaired Student’s t test. A reduction in growth was observed at 48 h, DMSO vs cytochalasin D or jasplankinolide (500 nm-1 µM) ****P < 0.0001; one-way ANOVA, DMSO vs CK-666, **P = 0.0021; unpaired Student’s t test. The growth further decreased at 72 h**.** DMSO vs cytochalasin D or jasplankinolide (500 nm-1 µM), ****P < 0.0001 and DMSO vs CK-666 ***P = 0.0001; unpaired Student’s t test. Compared with DMSO, 100 nM jasplankinolide did not affect parasite growth at 48 h (P = 0.6447) or 72 h (P = 0.5590). The data are presented as the mean ± SEM and were analyzed via one-way ANOVA or unpaired Student’s t tests; n = 3 biological replicates. **(C)** *P. berghei* blood cultures were treated with the inhibitors cytochalasin D (1 µM), jasplankinolide (250 nM) and CK-666 (300 µM). Counting of ring, trophozoite, and schizont stages in *P. berghei* cultures after treatment. There was no effect on the ring (cytochalasin D, P = 0.1605; jasplankinolide, P = 0.6896; and CK-666, P = 0.0693) or trophozoite stage (cytochalasin D, P = 0.8053; jasplankinolide, P = 0.1031; and CK-666 (P = 0.4832). The number of schizonts decreased significantly in all the treated groups (cytochalasin D, *P = 0.0257; jasplankinolide, **P = 0.0082; and CK-666, **P = 0.0012). The data are presented as the mean ± SEM and were analyzed via unpaired Student’s t tests; n = 3 biological replicates. **(D)** Asexual blood-stage propagation of control and KO parasites. The data are presented as the mean ± SEM, with no significant differences (P = 0.9998; Brown-Forsythe ANOVA). **(E)** Estimation of gametocytemia. The data are presented as the mean ± SEM with no significant differences (P = 0.7776; Brown-Forsythe ANOVA). **(F)** The male and female gametocyte ratios were comparable in WT and KO parasites. The data are presented as the mean ± SEM, with no significant differences (P = 0.9864; one-way ANOVA). Data are presented from two biological replicates, each with 5 mice per group in D, E, and F. **(G)** Western blot analysis of F-actin and G-actin in blood-stage parasites. Parasites were homogenized in F-actin stabilization buffer, followed by centrifugation to separate F-actin from the G-actin pool. Immunoblotting was performed with an anti-actin antibody to detect actin. Western blot quantitation of F-actin and G-actin in blood-stage parasites and data are presented as the F/G actin ratio. There was no difference between WT and Alp5 KO parasites (P = 0.5903; one-way ANOVA). **(H)** Comparison of exflagellation centers in WT and Alp5 KO parasites revealed no difference (P = 0.1274, Brown Forsythe and Welch ANOVA). Counts were checked from 10 random fields from three biological replicates. The data are presented as the mean ± SEM.

Since Alp5a and Alp5b are paralogous genes [44], we investigated their roles together. To understand the role of Alp5a and Alp5b in the parasite life cycle, we disrupted the gene in *P. berghei* via double crossover homologous recombination (S9A, S9B, S9E and S9F Fig). To restore gene function in the KO parasites, a complemented line was generated by reintroducing the Alp expression cassette (S9C, S9D, S9G, S9H and S9I Fig). We attempted to disrupt both genes simultaneously to check for redundancy in the functions of Alp5a and Alp5b. We applied two independent strategies for the double disruption of genes. First, we transfected an Alp5b-targeting construct into Alp5a KO schizonts. (S10A Fig). Twenty-four hours post-transfection, we observed a few parasites with red and green fluorescence, which were lost after WR drug selection. Next, we transfected *P. berghei* WT schizonts with Alp5a and Alp5b targeting constructs (S10B Fig). Parasites were selected using pyrimethamine drugs. We observed parasites with individual gene knockouts, but no double-deletion events were detected (Fig 3A). We treated *Plasmodium* blood-stage parasites with various inhibitors to further confirm the essential roles of actin and the Arp2/3 complex. We found that an Arp2/3 complex inhibitor (CK-666), an actin polymerization inhibitor (Cytochalasin D), and an actin polymerization inducer (Jasplakinolide) inhibited the growth and development of *P. falciparum* and *P. berghei* parasites (Figs 3B, 3C, S11A and S11B).

For phenotypic analysis of the Alp5a and Alp5b KO parasites, we assessed asexual blood-stage propagation. Both KO lines exhibited normal asexual replication and produced gametocytes at rates comparable to WT parasites (Fig 3D and 3E). Next, we examined the proportions of male and female gametocytes and found that they were comparable in WT and KO parasites (Fig 3F). We also analyzed the F-actin/G-actin ratio in the KO parasites after separating them by ultracentrifugation, which was comparable to that in the WT parasites (Fig 3G). Next, we analyzed microgamete formation. There was no significant difference in the number of exflagellation centers between KO and WT parasites (Fig 3H). These results indicate that the Alps are not individually required for actin nucleation or blood-stage development but are essential as a complex for parasite survival.

### Alp5s are essential for microgamete fertility, oocyst development, and malaria transmission

**Fig 4:**
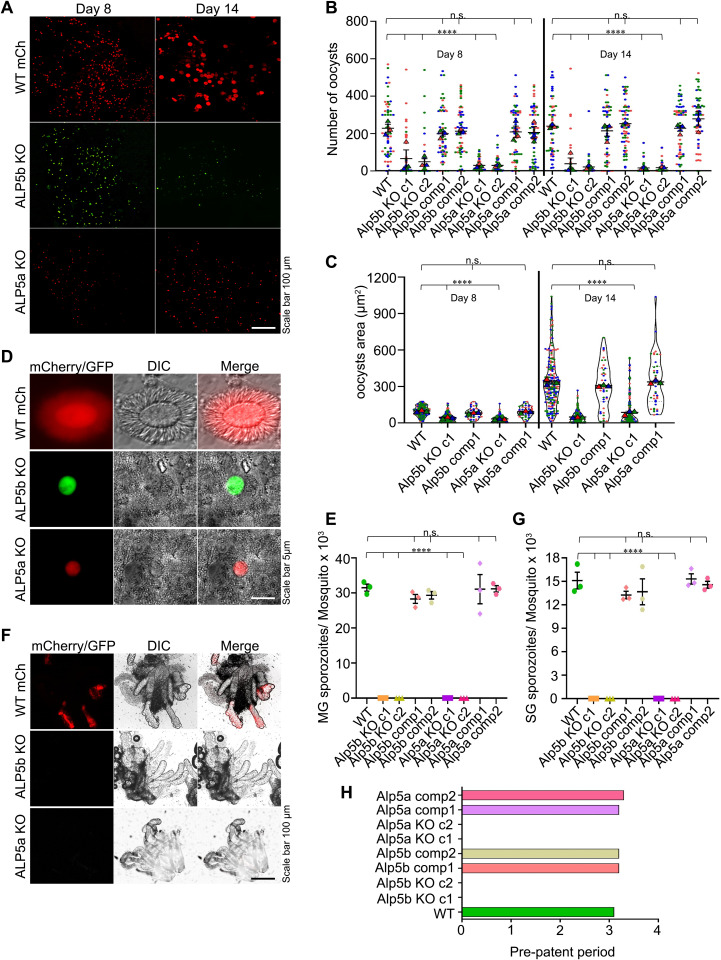
Alp5s are essential for oocyst development and malaria transmission. **(A)** Representative widefield fluorescence microscopy images of mosquito midguts showing oocysts on the indicated days post-infection. (**B**) Quantification of oocyst numbers. There was a significant difference in oocyst number between WT and KO parasites on days 8 and 14 (****P < 0.0001). In contrast, no differences were detected between the complement lines and WT on days 8 (P = 0.8721) and 14 (P = 0.1075). Sixty midguts from all the groups were dissected each day. (**C**) Determination of the oocyst area. The data from independent clones were pooled, and a significant difference was detected between the WT and KO lines on days 8 and 14 (****P < 0.0001), whereas no differences were detected between the complemented lines and the WT line on days 8 (P = 0.1058) and 14 (P = 0.8983). The Kruskal‒Wallis test was used to determine the significance. A total of 200 oocyst areas were measured for each of the WT, Alp5a KO, and Alp5b KO lines, and 40 oocyst areas for each of the corresponding complemented lines, on the indicated days. Data represent three independent biological replicates. The triangle represents the mean individual count in each experiment, and each dot represents an individual count. (**D**) Widefield fluorescence microscopy of WT oocysts undergoing sporogony. No sporogony was observed in Alp5a or Alp5b KO oocysts. (**E**) Midgut sporozoite count. The data revealed a significant difference between the WT and knockout lines (****P < 0.0001; one-way ANOVA). No difference was observed between the WT and complemented lines (P = 0.7737). The total number of mosquitoes dissected in each group was 95 (WT GFP), 235 (Alp5b c1), 235 (Alp5b c2), 95 (Alp5b comp1), 70 (Alp5b comp2), 260 (Alp5a c1), 245 (Alp5a c2), 80 (Alp5a comp1), and 85 (Alp5a comp2). Data are presented as the mean ± SEM from three independent biological replicates. (**F**) Widefield fluorescence microscopy reveals sporozoite-specific fluorescence in WT but not KO-infected salivary glands. (**G**) Salivary gland sporozoite count. The total number of mosquitoes dissected in each group was 85 (WT GFP), 118 (Alp5b c1), 170 (Alp5b c2), 70 (Alp5b comp1), 100 (Alp5b c2), 135 (Alp5a c1), 235 (Alp5a c2), 70 (Alp5a comp1), and 70 (Alp5a comp2). The data revealed a significant difference between the WT and knockout lines (****P < 0.0001; one-way ANOVA). No difference was detected between the WT and complemented lines (P = 0.5317; one-way ANOVA). Data are presented as the mean ± SEM from three independent biological replicates. (**H**) Transmission of sporozoites to mice via mosquito bites (n = 5 mice per group) was assessed. Infection was not detected in mice bitten by mosquitoes infected with the KO parasites. The data represent pooled results from two independent biological replicates.

To analyze the development of Alp5 KO parasites in mosquitoes, we allowed mosquitoes to feed on the infected mice to facilitate transmission. We observed oocyst development on day 14 post-bloodmeal and found a significant reduction in oocyst size in both types of KO parasites (Figs 4A and S12A). Next, we analyzed the size and number of oocysts from days 8–18 post-feeding. The size and number of oocysts were significantly reduced in KO parasites. Interestingly, the number of oocysts decreased over time, with only a few remaining by day 18 (Figs 4B, 4C, S12B and S12C). Additionally, oocyst size did not increase, and sporogony was abolished in the KO parasites (Fig 4D). We crushed the midgut and salivary glands to estimate the sporozoite numbers. The sporozoites were completely absent in the Alp5 KO parasites (Fig 4E, 4F and 4G). All the KO-infected mosquitoes failed to transmit malaria to the mice in the biteback experiment (Fig 4H and Table 1). The complemented parasite lines successfully completed all developmental stages, confirming that the observed phenotype resulted from the deletion of the Alp5 gene (Fig 4). These results demonstrate that Alp5 is essential for oocyst development and sporogony, and that its deletion blocks malaria transmission.

**Table 1:** In vivo infectivity of Alp5 KO parasites in C57BL/6 mice. Sporozoites were inoculated into C57BL/6 mice via mosquito bites or intravenous injection. The presence of parasites in the blood was confirmed via a Giemsa-stained blood smear.

Experiment	Parasites	Number of mosquitoes/mouse	Mice positive/Mice infected	Pre-patent period (days)
**1**	WT-mCherry	50	5/5	3.2
	Alp5b KO c1	50	0/5	NA
	Alp5b KO c2	50	0/5	NA
	Alp5b comp1	50	5/5	3.2
	Alp5b comp2	50	5/5	3.4
	Alp5a KO c1	50	0/5	NA
	Alp5a KO c2	50	0/5	NA
	Alp5a comp1	50	5/5	3.4
	Alp5a comp2	50	5/5	3.2
**2**	WT	50	5/5	3
	Alp5b KO c1	50	0/5	NA
	Alp5b KO c2	50	0/5	NA
	Alp5b comp1	50	5/5	3.2
	Alp5b comp2	50	5/5	3
	Alp5a KO c1	50	0/5	NA
	Alp5a KO c2	50	0/5	NA
	Alp5a comp1	50	5/5	3
	Alp5a comp2	50	5/5	3.4
**Experiment**	**Parasites**	**Number of sporozoites injected**	**Mice positive/Mice** **injected**	**Prepatent period (days)**
**1**	WT	5,000	5/5	3.2
	WT-mCh × Alp5b KO	5,000	5/5	3.4
	WT-GFP × Alp5a KO	5,000	5/5	3.6
	Pb47 × Alp5b KO	5,000	5/5	3.6
	Pb47 × Alp5a KO	5,000	5/5	3.6

**Fig 5:**
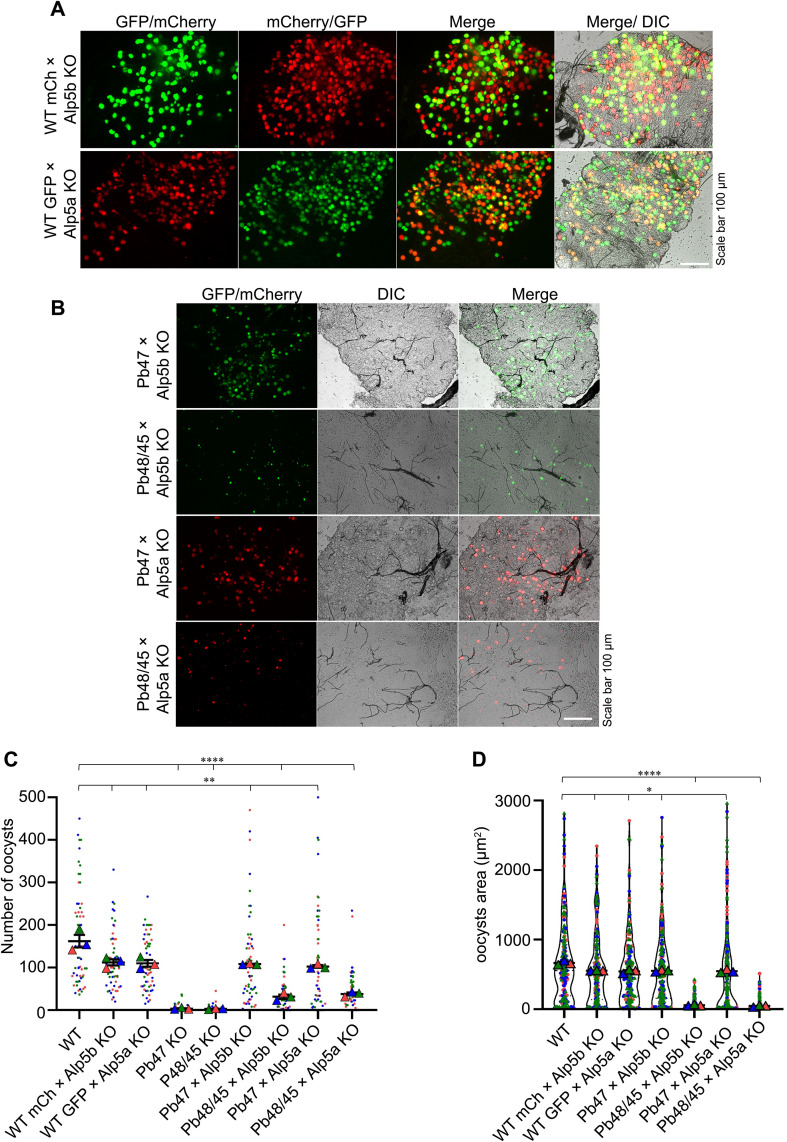
Genetic cross revealed a defect in the male gamete in Alp5 KO parasites. **(A)** Widefield fluorescence microscopy of oocysts at day 15 post-feeding following genetic crosses between WT-mCherry×Alp5b KO or WT-GFP × Alp5a KO parasites. **(B)** Genetic crosses of Alp5 KO parasites with male or female gamete-defective lines. The phenotype was restored in the KO lines crossed with the female gamete-defective lines. Genetic crosses with the male gamete-defective lines did not restore the KO phenotype. **(C)** Quantification of oocysts on day 15. Sixty-two midguts from each group were dissected and observed. Significant differences were observed in WT vs WT-mCherry×Alp5b KO (**P = 0.0038), WT vs WT-GFP × Alp5a KO (**P = 0.0019), WT vs Pb47 KO (****P < 0.0001), WT vs Pb48/45 KO (****P < 0.0001), WT vs Pb47 KO × Alp5b KO (**P = 0.0071), WT vs Pb48/45 KO × Alp5b KO (****P < 0.0001), WT vs Pb47 KO × Alp5a KO (**P = 0.0050), and WT vs Pb48/45 KO × Alp5a KO (****P < 0.0001). **(D)** Oocyst area on day 15 post-infection. A total of 210 oocyst areas were quantified in each group. Significant differences were observed in WT vs WT-mCherry×Alp5b KO (*P = 0.0377), WT vs WT-GFP × Alp5a KO (*P = 0.0203), WT vs Pb47 KO × Alp5b KO (*P = 0.0334), WT vs Pb48/45 KO × Alp5b KO (****P < 0.0001), WT vs Pb47 KO × Alp5a KO (*P = 0.0435), and WT vs Pb48/45 KO × Alp5a KO (****P < 0.0001). Unpaired Student’s t test was used to determine the statistical significance. The data were pooled from three independent biological replicates and are presented as the mean ± SEM in panels C and D.

After the essential roles of Alp5a and Alp5b in oocyst development and malaria transmission were established, the formation of ookinetes was subsequently analyzed. We found no difference in ookinete number or morphology between WT and KO parasites (S13A and S13B Fig). A genetic cross was performed between the KO and WT parasites to analyze sex-specific defects in the KO parasites. On day 15, post-feeding, we dissected the midgut, and the development of oocysts was observed. Genetic crosses with WT parasites restored the KO parasite’s phenotype (Fig 5A), indicating a defect at the gamete stage. *Plasmodium* inherits many defects during mosquito stage development from one sex [11]. To investigate whether the functions of Alp5a and Alp5b are sex specific, we generated male and female gamete defective lines by knocking out the Pb48/45 [54] and Pb47 genes [55], respectively (S14 Fig). Next, we crossed KO parasites with gamete defective lines (Pb47 × Alp5b KO, Pb48/45 × Alp5b KO, Pb47 × Alp5a KO, Pb48/45 × Alp5a KO). Crossing Alp5 KO with Pb47 KO parasites resulted in robust oocyst formation, indicating that Alp5 KO parasites produce fertile female gametes (Figs 5B and S15). In contrast, the Alp5 KO × Pb48/45 KO cross showed no change in oocyst development, indicating defects in microgamete function in Alp5 KO parasites (Figs 5B and S15). Following genetic cross-experiments, oocysts were quantitatively assessed by counting and measuring their surface area, and sporogony progression was monitored. Sporogony of the restored oocysts proceeded normally (Fig 5C and 5D). On day 19 post-blood feeding, dissection of mosquito salivary glands revealed normal sporozoite loads in all experimental groups except the Alp5-KO lines crossed with a male gamete-defective parasite line (S16A and S16B Fig). The obtained salivary gland sporozoites were checked for infectivity in vivo and in vitro. Sporozoites were intravenously injected into C57BL/6 mice, and the onset of the pre-patent period was monitored starting from day three post-infection (Table 1). Groups with restored salivary gland sporozoite loads successfully infected hepatocytes and progressed normally to the blood stage (S17 Fig). Together, these results demonstrated that Alp5s play male-specific roles in gamete fertility.

### Alp5s regulate DNA segregation during male gametogenesis

**Fig 6:**
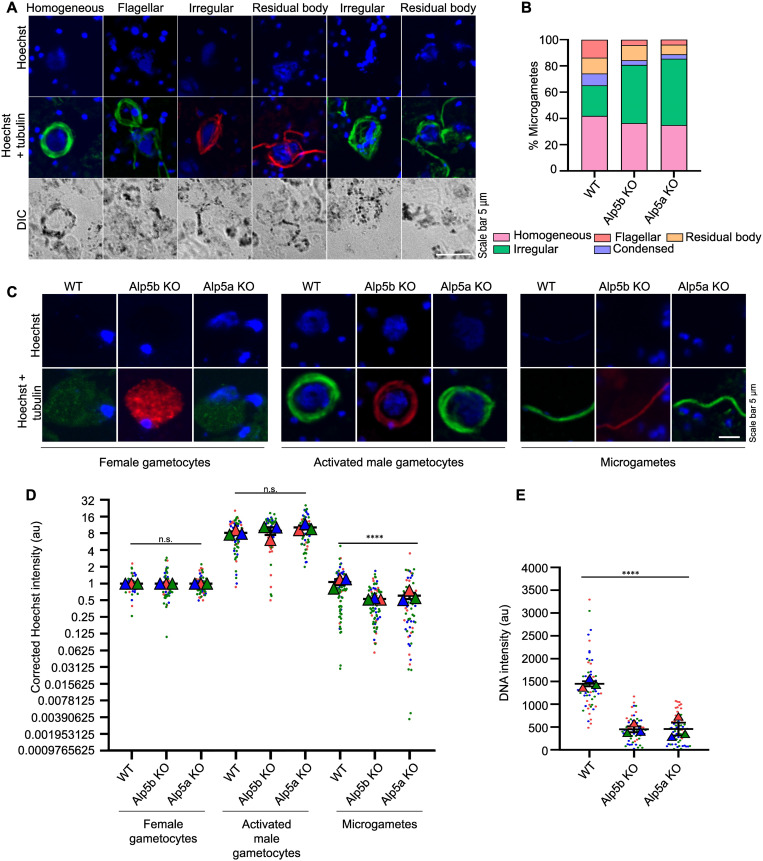
Impaired DNA segregation in Alp5 KO parasites. **(A)** Gametocytes were activated and fixed at 15 mpa (minutes post-activation). DNA was stained with Hoechst, and an anti-tubulin antibody was used to identify activated male gametocytes. Images were acquired using a confocal microscope. **(B)** Different forms of microgametes were identified based on quantification of DNA localization patterns in activated male gametocytes. A total of 167 (WT), 192 (Alp5b KO), and 295 (Alp5a KO) male gametocytes were analyzed from three biological replicates. **(C)** Confocal IFA images of female gametocytes, activated male gametocytes and microgametes. Activated male gametocytes and microgametes were identified by anti-tubulin antibody staining. The female gametocytes were identified via diffuse tubulin staining. DNA was stained with Hoechst. **(D)** We quantified the DNA content of activated male and female gametocytes as well as microgametes, normalizing all measurements to the mean DNA content of female gametocytes from the same slide. Analysis included 70 female gametocytes, alongside male gametocytes from WT (68), Alp5b KO (69), and Alp5a KO (65) lines. Microgametes were similarly assessed with sample sizes of 92 (WT), 89 (Alp5b KO), and 64 (Alp5a KO). The data were comparable between WT and Alp5 KO lines in terms of the number of female gametocytes (P = 0.3126) and activated male gametocytes (P = 0.148). The DNA content significantly differed between WT and Alp5 KO microgametes (****P < 0.0001). Data from three independent biological replicates were analyzed using the Kruskal–Wallis test to assess statistical differences among groups. **(E)** Quantification of the zygote DNA content (a.u., arbitrary unit). We detected significant differences among the WT, Alp5a KO and Alp5b KO parasites (****P < 0.0001). The DNA content of 60 zygotes per group was analyzed. Data from three independent biological replicates were analyzed using Student’s t test to assess statistical differences.

**Fig 7:**
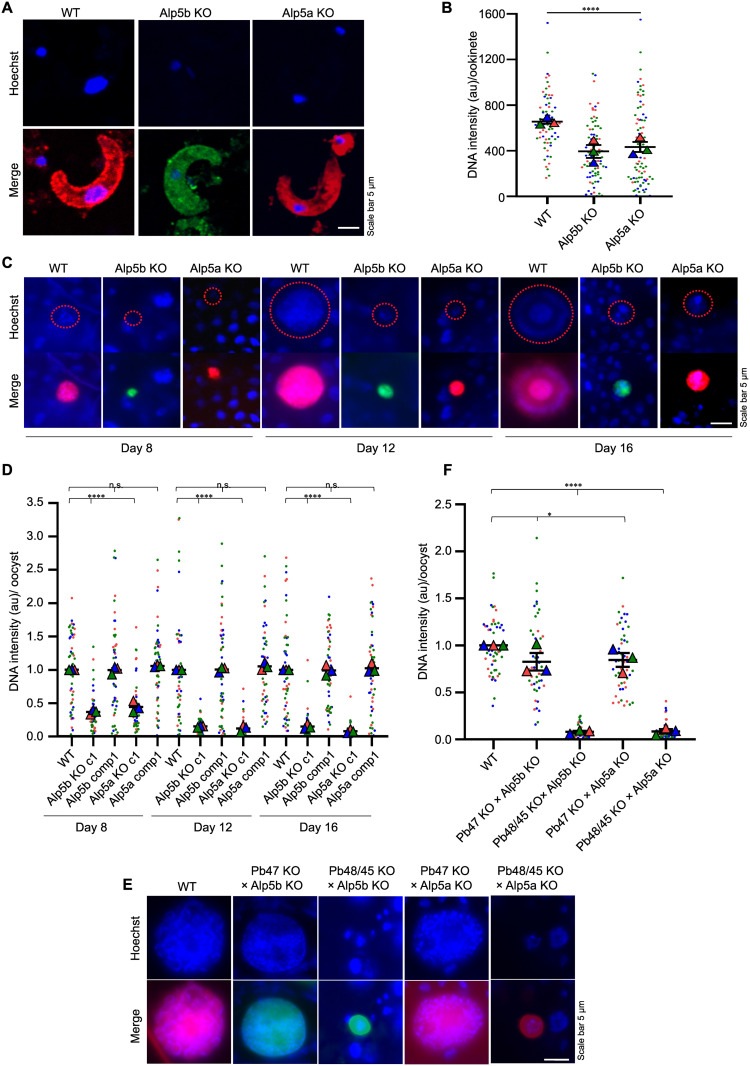
DNA content is reduced in the ookinete and oocyst of Alp5 KO parasites. **(A)** Representative confocal immunofluorescence images of ookinetes. Compared with WT ookinetes, Alp5 KO ookinetes presented a lower DNA content. **(B)** Quantification of ookinete DNA content (a.u., arbitrary unit). We detected significant differences between WT and Alp5 KO parasites (****P < 0.0001). The DNA contents of 70 (WT), 83 (Alp5b), and 83 (Alp5a) ookinetes were quantified. Data from three independent biological replicates were analyzed using the Kruskal–Wallis test to assess statistical differences among groups. **(C)** Widefield fluorescence microscopy images of Hoechst-stained oocysts showing the DNA content on different days. **(D)** The average DNA intensity of 50 oocysts was significantly different between WT and Alp5 KO parasites on days 8 (****P < 0.0001), 12 (****P < 0.0001) and 16 (****P < 0.0001). The DNA content was comparable between WT, ALP5b comp and ALP5a comp on day 8 (P = 0.7347), day 12 (P = 0.6874), and day 16 (P = 0. 8970). Data from three independent biological replicates were analyzed using the Kruskal–Wallis test to assess statistical differences among groups. **(E)** Widefield fluorescence microscopy images of Hoechst-stained oocysts illustrate the DNA content after the genetic crosses. The DNA content was restored in Alp5 KO parasites crossed with female gamete-defective lines. In contrast, genetic crosses with male gamete-defective lines did not rescue the DNA content. **(F)** The DNA content of 50 oocysts in Alp5 KO parasites was quantified and normalized to the mean DNA intensity of WT oocysts. A significant difference was observed in WT vs Pb47 KO × Alp5b KO (*P = 0.0330), WT vs Pb48/45 KO × Alp5b KO (****P < 0.0001), WT vs Pb47 KO × Alp5a KO (*P = 0.0172), and WT vs Pb48/45 KO × Alp5a KO (****P < 0.0001). Data from three independent biological replicates were analyzed using Student’s t test to assess statistical differences.

The male gametocyte undergoes three rapid rounds of DNA division during male gametogony, reaching an 8 N state. After division, this DNA distributed to the eight flagellar microgametes coincided with axoneme formation. To determine the defect in male gametogony, activated gametocytes were immunostained with an anti-tubulin antibody to identify activated microgametes and free microgamete. On the same slide, a single nucleated cell exhibiting a diffuse signal was identified as a female gametocyte (macrogamete). Despite no observable differences in the number of activated male and female gametes, we identified distinct patterns of DNA allocation within activated gametocytes. The DNA distribution was homogenous, flagellar and condensed in the WT parasites, with enlarged activated gametocyte nuclei. However, the DNA was residual and irregularly distributed in the Alp5 KO parasites (Fig 6A). The percentage of irregularly distributed Alp5a KO and Alp5b KO cells was greater than that of WT cells (Fig 6B). We found no difference in the DNA content of activated male gametocytes (enlarged nuclei coinciding with axonemes) or female gametocytes/gametes (single nuclei with diffuse tubulin signals). The microgametes from the KO showed a significant reduction in DNA content—approximately 50% less compared to the wild type WT. (Fig 6C and 6D). The DNA was quantified via the corrected total cell fluorescence (CTCF) method via ImageJ software. The reduced DNA content in the microgametes of Alp5 KO parasites did not affect the morphology of zygotes or ookinetes. Next, we checked the DNA content in zygotes and ookinetes, which was significantly reduced in Alp5 KO parasites (Figs 6E, 7A and 7B). The reduced DNA content in the ookinete did not affect its gliding ability (S13C Fig). Next, we analyzed the DNA within the oocysts at various time points using Hoechst staining. The stained oocysts were then imaged, and their DNA content was quantified using Fiji software. The DNA content in the KO oocysts was significantly reduced, indicating impaired development. In contrast, the DNA content in the WT and complemented lines was comparable (Fig 7C and 7D). The DNA content of oocysts derived from crosses between KO lines and female gamete-defective lines was comparable to that observed in WT. In contrast, crosses between KO lines and male gamete-defective lines showed no significant change in oocyst DNA content (Fig 7E and 7F). These results suggest that Alp5 proteins are essential for DNA segregation during male gametogenesis. Although Alp5 KO parasites can undergo meiosis and ookinete formation despite having reduced DNA content, they are arrested at the oocyst stage.

## Discussion

**Fig 8:**
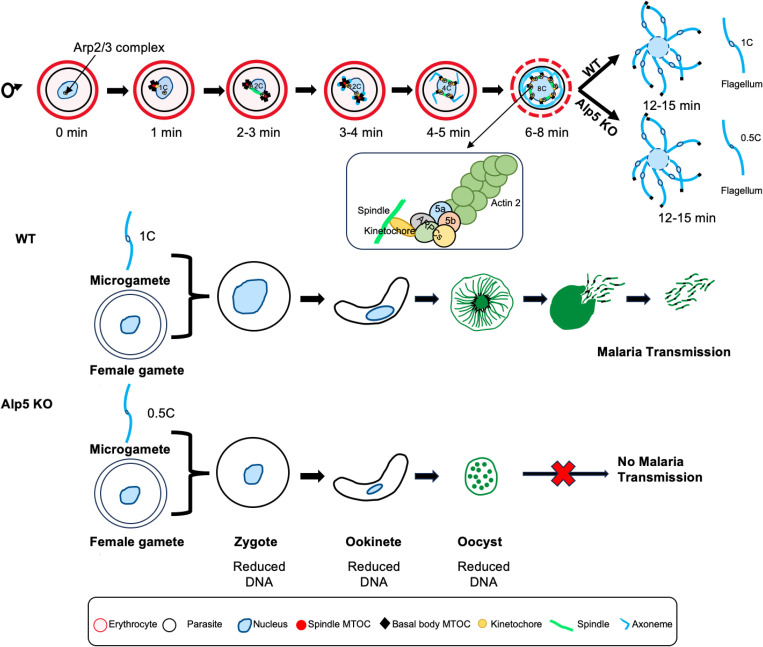
Model of non-canonical Arp2/3 complex-mediated actin nucleation during male gametogony and its impact on parasite development. During male gametogony, actin stabilizes the endomitotic spindle assembly, ensuring proper chromosomal segregation. Parasites deficient in Alp5 exhibit reduced DNA content transitioning from microgametes to oocysts. This compromised genomic integrity in the male lineage likely impairs DNA replication, leading to defective oocyst development and maturation.

The actin cytoskeleton is involved in a plethora of cellular functions [56]. The rate-limiting step in actin filament assembly is the nucleation step. One of the major actin-nucleating factors in cells is the seven subunit Arp2/3 complex. The Arp2/3 complex is highly conserved in all eukaryotes [42,43,46]. In this study, we investigated the functions of the human Arp2- and Arp3-related *Plasmodium* proteins Alp5a and Alp5b, which revealed that these proteins have distinct cellular functions in DNA segregation during male gametogenesis. A study recently identified a non-canonical Arp2/3 complex in *Plasmodium* that consists of only five subunits [40]. We found that the Arp2/3 complex subunits, Alp5a and Alp5b, localize from the nucleus to a microtubule-rich structure, likely the spindle or axoneme and remain in the residual body after exflagellation. Parasites lacking Alp5, form subhaploid microgametes that are still capable of fertilization, giving rise to functional zygotes and motile ookinetes. However, Alp5 knockout parasites exhibit a delayed-death-like arrest at the oocyst stage, ultimately resulting in a complete block of transmission within the mosquito vector (Fig 8).

Alp5s are predominantly expressed in the nucleus and are localized from the nucleus to a microtubule-rich structure, likely the spindle or axoneme. We found that Alp5s colocalize with F-actin and that Alp5 KO parasites exhibit defects in DNA segregation and microgamete fertility. Like human counterparts, *P. berghei* Alp5a and Alp5b interact with each other. It has been reported that actin 2 is present in male gametocytes, female gametes, and zygotes [25,57] and that it is associated with the nucleus in the male gametocyte and in the zygote. Although actin 2 is predominantly found in the nucleus, its signals are also detected in the cytoplasm [23]. Similarly, we detected the Alp5s signal in the cytoplasm, indicating its additional role beyond nuclear function. Despite the loss of either Alp5a or Alp5b individually, F-actin to G-actin ratios in knockout parasites remained comparable to WT. The simultaneous indispensability of Alp5a and Alp5b, together with the observed inhibition of *P. berghei* and *P. falciparum* blood-stage development upon treatment with an Arp2/3 complex inhibitor, supports a functional role for the Arp2/3 complex during the erythrocytic stage of the parasite life cycle. The STRING database revealed the interaction of *P. berghei* Alp5 with Formin 2. We hypothesize that in the absence of the Alp5 subunit, the parasite relies on Formin 2 to maintain cytoskeletal regulation. The malaria parasite Formin regulates actin polymerization, and the parasite uses polymerized actin throughout its lifecycle. Formin 1 is essential for invading merozoites, and Formin 2 is critical for efficient cell division in *P. falciparum* [36]. Formin inhibitors block multiple asexual and sexual parasite stages of development [36]. Given the cytosolic localization of Formin 2 [36], it is plausible that Formin 2 and the Arp2/3 complex function cooperatively to nucleate actin. However, this possibility remains to be experimentally tested. Another possible compensatory mechanism by which a subset of ABPs may substitute for the missing components of the Arp2/3 complex during blood-stage development. Notably, proteins such as profilin [39], capping proteins [37,38], and ADF/cofilin [33,34] are known to modulate actin dynamics in a tightly regulated manner. It is tempting to speculate that these ABPs may function in concert with Alp5 to nucleate actin in the absence of a fully assembled Arp2/3 complex. This potential compensation may reflect a broader evolutionary adaptation to the unique intracellular environment encountered during erythrocytic stages. Malaria parasites encode two actin isoforms, actin 1 and actin 2. Actin 1 is indispensable during *Plasmodium* blood stage development [26]. Actin 2 is dispensable in blood stage but has essential functions in male gametogenesis and oocyst development [25,27]. Actin 2 mainly localizes to the nuclear spindle and, to some extent, to the cytoplasm and actin 2 mutant male gametocytes fail to exflagellate [23,27]. Attempts to complement actin 2 function with actin 1 partially restored male gametogenesis, resulting in the formation of oocysts, but these oocysts failed to form sporozoites [25]. The inability of actin 1 to complement the function of actin 2 suggests that the Arp2/3 complex nucleates actin 2 without associating with Formin 2.

We observed defects in microgamete integrity in Alp5 KO parasites through genetic crossing experiments. The sporogony in rescued oocysts proceeded normally, and the resulting sporozoites were capable of successfully infecting both salivary glands and mammalian hosts. The lack of Alp5 expression in the sporozoites and liver stage indicates that the Arp2/3 complex plays no role during these stages. Therefore, the primary function of the Arp2/3 complex is likely the nucleation of actin 2. We found that the DNA content of microgametes significantly differed between WT and Alp5 KO parasites. The localization of Alp5s to the axoneme suggests that Arp2/3 act at the level of DNA segregation. A recent report revealed that the Arp2/3 subunit ARPC1 localizes to the endomitotic spindles interacts with the kinetochore protein AKiT7, and acts at the kinetochore-spindle interphase [40]. They found a decrease in the DNA content of microgametes in ARPC1, Alp5b, or ARPC2 KO parasites. ARPC1 KO parasites fail to form and maintain the eight kinetochore foci during DNA condensation and subsequent exflagellation [40]. Together with these results, we speculate that actin polymerization is required to stabilize the attachment of kinetochores to the spindle during axonemal movement. In Alp5 KO parasites, a greater proportion of irregular microgametes indicates a defect in DNA segregation.

Alp5 KO parasites are able to form oocysts but fail to undergo sporogony, exhibiting a delayed death phenotype. This resembles the phenotype observed in *P. berghei* lacking end-binding protein 1 (EB1) [58]. EB1 links kinetochores to the spindle during male gametogenesis, and its absence results in a similar delayed death phenotype [58]. Other mutant parasites with early oocyst development defects include those lacking the formin-like protein MISFIT [59], aurora-related kinase-2 (Ark2) [58], and the microtubule motor kinesin-8X [60]. Oocyst development involves endomitosis, resulting in the formation of a single multinucleated oocyst [61]. There’s no direct link between endomitosis and the Arp2/3 complex, it’s very reasonable to hypothesize that dysfunctional Arp2/3 activity could contribute indirectly to endomitosis through its role in actin dynamics and cytokinesis. Although Alp5 KO parasites form oocysts containing a complete genome from the female gamete, the compromised male genome—potentially with reduced DNA content—may impair DNA replication during oocyst development. It remains unclear whether this phenotype is solely due to microgamete defects or whether the Arp2/3 complex, of which Alp5 is a part, plays a direct role in oocyst development. Further investigation is required to clarify this.

In conclusion, we show here that *Plasmodium* Alp5s are essential for malaria parasite transmission to mosquitoes. Although neither Alp5a nor Alp5b alone is essential for the parasite’s asexual blood stage development, our data demonstrate that both proteins play indispensable roles during male gametogenesis. Disrupting their function may impair microgamete formation and thus interrupt malaria transmission. Further investigation is required to elucidate the precise roles of the Arp2/3 complex and the mechanisms governing cell division in microgametes.

## Materials and methods

### Ethics statement

All animal experiments were conducted in accordance with relevant guidelines and regulations and were approved by the Institutional Animal Ethics Committee of the CSIR-Central Drug Research Institute, India (IAEC Reference Nos: IAEC/2018/3 to IAEC/2023/15). Human red blood cells (RBCs) used for parasite culture were obtained under protocols approved by the Institutional Ethics Committee – Human Research, CSIR-Central Drug Research Institute (Approval No. CDRI/IEC/2017/A4).

### Bioinformatics analysis

The protein sequences of *Plasmodium berghei* Alp5a and Alp5b were retrieved from PlasmoDB (https://plasmodb.org). The amino acid sequences were queried via NCBI protein BLAST (BLAST P) against model organisms. Owing to the unavailability of experimentally determined structures, the protein structures of PbAlp5a and PbAlp5b were predicted via AlphaFold 2 [62,63]. The quality of the predicted structure was assessed via SAVES v6.0 (https://saves.mbi.ucla.edu/). Further experimental high-resolution structures of *P. falciparum* actin 1 (6I4H with the resolution of 1.40 Å) and *P. berghei* actin 2 (6I4M with the resolution of 1.87 Å) were retrieved from the RCSB PDB database. To assess structural similarity and protein folding, the structure of Alp5a was superimposed onto the known human Arp3 protein structure, and Alp5b was superimposed onto the human Arp2 structure (human Arp2:6YW6 and Arp3:6UHC proteins from the PDB database https://www.rcsb.org/). Structural alignment and superposition were performed using pairwise alignment, employing the super command with an outlier rejection cutoff score of 2.0. The amino acid sequences of Alp5a and Alp5b were submitted to the STRING database to investigate potential interactions between these proteins [64]. To predict potential protein-protein interactions, docking studies were performed using the ClusPro server [65]. The workflow involves three primary steps: rigid-body docking using the PIPER algorithm to generate candidate conformations; clustering of low-energy poses based on structural similarity to identify the most representative complexes; and energy minimization to refine the selected models. Docked complexes were ranked according to cluster size and the weighted energy score of the lowest-energy member within each cluster. Subsequently, to predict the ternary complex, interaction studies were repeated using AlphaFold3 [66]. The top-ranking models were downloaded and visualized using PyMOL [67].

### Parasites, mosquitoes and mice

*Plasmodium berghei* ANKA (MRA 311) and *P. berghei* ANKA GFP (MRA 867 507 m6cl1) were obtained from BEI Resources, USA. *Anopheles stephensi* mosquitoes were maintained in a walk-in environmental chamber at 28 °C, 80% relative humidity, and a 12:12 h light:dark cycle. Adults were provided with 10% (w/v) sucrose solution on cotton pads. Female *Anopheles* mosquitoes were allowed to probe and feed on Swiss mice to obtain a blood meal necessary for egg development. The parasites were transmitted to mosquitoes as previously described [68]. The infected mosquitoes were maintained in a walk-in environmental chamber set at 19 °C with 80% relative humidity and a 12-hour light/dark cycle. Swiss albino and C57BL/6 mice (6–8 weeks old) were used in this study. Chloroquine-sensitive *P. falciparum,* strain 3D7 (MRA-151, BEI Resources, USA) was used to assess the effects of the inhibitors. Parasites were cultured in human RBCs at 2% haematocrit in HEPES-modified RPMI-1640 medium (Sigma Aldrich, USA) supplemented with 1% glucose, 0.2% sodium bicarbonate, 100 µM hypoxanthine (Sigma-Aldrich #Cat. H9636), gentamycin (Gibco #Cat. 15750–060), (25 μg/ml) and 0.5% Albumax II (Gibco #Cat. 11021–037). The parasites were regularly synchronized with 5% D-sorbitol [69]. Human liver hepatocellular carcinoma (HepG2) cells (ATCC) were cultured in DMEM (Sigma-Aldrich #Cat. D5648) enriched with 10% FBS (Biological Industries #Cat. 04–121-1A), 0.2% NaHCO_3_ (Sigma-Aldrich #Cat. S5761), 1% sodium pyruvate (Genetix #Cat. CC4016), and 1% penicillin‒streptomycin (Gibco #Cat. 15140–122) maintained at 37°C with 5% CO2.

### Generation of Alp5b knockout and complemented parasite lines

To delete the Alp5b gene, the wild-type locus was replaced with a targeting cassette via double crossover homologous recombination. Two fragments, F1 (0.545 kb) and F2 (0.6 kb), were amplified from the 5’ and 3’ UTR regions via the primer sets 1611/1612 and 1613/1614, respectively. Fragments F1 and F2 were subsequently cloned into the pBC-GFP-hDHFR-yFCU vector at *Sal*I and *Not*I/*Asc*I, respectively. The final targeting cassette was separated from the vector backbone via digestion with *Xho*I/*Asc*I and transfected into *P. berghei* ANKA schizonts as described previously [70]. Drug-resistant parasites that appeared after pyrimethamine selection were observed for GFP fluorescence. The genomic DNA was isolated from the GFP expressing drug-resistant parasites, and correct 5’ and 3’ site-specific integration was confirmed by PCR via the primer sets 1665/1225 and 1666/1215, respectively. The clonal lines were obtained by limiting dilution of the parasites and injecting them into Swiss mice. The clonal KO lines were confirmed via the primers 1671/1672. The Alp5b complemented line was generated by reintroducing the ORF via double crossover homologous recombination. A fragment encompassing the 5’UTR, ORF and 3’UTR was amplified via the primer set 1611/1614 and transfected into Alp5b KO schizonts. The transfected parasites were negatively selected with 5-fluorocytosine (Sigma-Aldrich #Cat. F7129) as previously described [71]. Gene restoration was confirmed by PCR via the primers 1671/1672.

### Generation of Alp5a knockout and complemented parasite lines

To disrupt the Alp5a locus, two fragments, F3 (0.59 kb) and F4 (0.52 kb), were amplified via the primer sets 1895/1896 and 1897/1898, respectively. Fragments F3 and F4 were sequentially cloned into a pBC-mCherry-tgDHFR vector at *Kpn*I/*Cla*I and *Not*I/*Asc*I, respectively. The plasmid was linearized via *Kpn*I/*Asc*I and transfected into *P. berghei* ANKA schizonts. Genomic DNA was isolated from mCherry expressing pyrimethamine-resistant parasites, and 5’ and 3’ site-specific integration was confirmed by diagnostic PCR via primers 2089/1225 and 1913/2090, respectively. Clonal lines were obtained as described above and confirmed via the primers 2091/2092. To restore gene function, fragment F5 encompassing the Alp5a-expressing cassette (5’UTR + ORF + 3’UTR) was amplified via primers 2268/2269 and subsequently cloned into pBC-P230-mCherry-hDHFR at *Cla*I and *EcoR*I. The linearized cassette was transfected into Alp5a KO schizonts, which were selected via the WR99210 drug (Sigma-Aldrich #Cat. SML2976) as previously described [72]. Correct 5’ and 3’ site-specific integration was confirmed by diagnostic PCR via primer sets 2270/2271 and 1215/2272, respectively. The restoration of the ORF was confirmed via the primer set 2091/2092. The primer sequences are given in S1 Table.

### Attempts to generate Alp5a/Alp5b double knockout parasites

Two strategies were employed to generate Alp5a/Alp5b double KO parasites. In the first strategy, the linearized cassette of Alp5b was transfected into Alp5a KO schizonts, which were selected with the WR99210 drug. In the second strategy, Alp5b and ALP5a targeting cassettes were cotransfected into *P. berghei* ANKA schizonts, which were selected with pyrimethamine (Sigma-Aldrich #Cat. 46706).

### Generation of Alp5a and Alp5b transgenic parasites

For Alp5b tagging with 3XHA-mCherry, two fragments, F6 (0.54 kb) and F7 (0.60 kb), were amplified via the primer sets 1675/1676 and 1677/1614 and cloned into the pBC-3XHA-mCherry-hDHFR vector at *Xho*I/*Bgl*II and *Not*I/*Asc*I, respectively. For Alp5a tagging with 3XHA, two fragments, F8 (0.61 kb) and F4 (0.52 kb), were amplified via the primers 2128/2129 and 1897/1898 and cloned into the pBC-3XHA-hDHFR vector at *Xho*I/*Bgl*II and *Not*I/*Asc*I, respectively. The vector was linearized via *Xho*I/*Asc*I and transfected into *P. berghei* ANKA schizonts as described above. Correct 5’ and 3’ site-specific integration was confirmed by diagnostic PCR via the primers 1724/1392 and 1215/1666 for Alp5b and 2130/1218 and 1215/2090 for Alp5a. The primer sequences are given in S1 Table.

### Generation of gamete-defective lines

The 6-cysteine proteins, P47 (PBANKA_1359700) and P48/45 (PBANKA_1359600) targeting constructs for generating female and male gamete-defective lines, respectively, were obtained from the PlasmoGEM resource (https://plasmogem.umu.se/pbgem/) [73]. The plasmids were linearized via *Not*I, transfected into *P. berghei* ANKA schizonts and selected with pyrimethamine. Genomic DNA was isolated from drug-resistant parasites, and site-specific integration of the P47 and P48/45 targeting cassettes was confirmed by diagnostic PCR via primers 2232/2035 and 2229/2036, respectively. Clonal lines were obtained by limiting dilution of the parasites, and the absence of P47 and P48/45 ORFs was confirmed via primers 2233/2234 and 2230/2231, respectively. The primer sequences are given in S1 Table.

### Asexual blood-stage propagation and gametocyte development

Two groups of Swiss mice (5 mice/group) were injected intravenously with 200 µl of blood containing 0.5% parasitemia. Blood growth and gametocyte development were monitored via Giemsa-stained blood smears.

### Analysis of parasite development in mosquitoes

Swiss mice were injected with WT or KO parasites as described above. The mice positive for gametocytes were anesthetized and placed in starved mosquito cages for a blood meal. To analyze the different developmental stages of parasites, mosquitoes were dissected under a stereo-zoom microscope. Blood boluses were collected 10–20 min or 18–22 h postfeeding to analyze gametes and ookinetes, respectively, and slides were prepared for live imaging as previously described [71]. The blood boluses was crushed and diluted, and the ookinetes were counted via a hemocytometer. Mosquito midguts were collected on days 8–18 to determine the oocyst numbers and areas. The oocyst area was determined via Fiji software. Midgut and salivary gland sporozoites were enumerated on days 14 and 19–22 post-blood meal, respectively, as previously described [72].

### Genetic cross

A genetic cross between the mutant and WT lines was performed. For this purpose, blood was collected from the mice infected with WT or KO parasites and mixed at a ratio of 1:1. Two hundred microliters of blood with 0.5% parasitemia was injected into a group of Swiss mice. Mosquitoes were allowed to probe for a blood meal in infected mice. Mosquitoes were dissected on days 15 and 19–22 after a blood meal to observe oocysts and salivary gland sporozoites, respectively.

### Gametocyte purification and activation

Gametocytes were purified using a 50% Nycodenz gradient. Swiss mice were pre-treated with phenylhydrazine (6 mg/ml; Sigma-Aldrich, Cat. #114715) for three consecutive days to induce reticulocytosis, and subsequently injected intraperitoneally with 200 µl of blood containing 1% parasitemia. After 3–4 days, the presence of gametocytes was confirmed by Giemsa-stained blood smears. Infected blood was collected into RPMI medium, layered onto a 50% Nycodenz gradient, and centrifuged at 200 × g for 20 minutes. The gametocyte-rich layer appearing at the interphase was carefully collected and washed 2–3 times with PBS to remove residual Nycodenz. The purified gametocytes were activated using an exflagellation medium composed of 25 mM HEPES (Sigma-Aldrich, Cat. #83264) and 100 µM xanthurenic acid (Sigma-Aldrich, Cat. #D120804), supplemented with RPMI medium (Sigma-Aldrich, Cat. #23400–013) at pH 8.0. Parasite stages were observed either live or after fixation with 4% paraformaldehyde (PFA; Sigma-Aldrich, Cat. #HT5012).

### Exflagellation centres

An exflagellation assay was conducted by incubating 10 µl of purified gametocytes with 40 µl of exflagellation medium at 20°C for 5–10 minutes. The exflagellation centres were observed under a phase contrast microscope at 100x magnification and counted per field.

### Ookinete motility assay

Blood boluses were isolated, diluted, and mixed with an equal volume of Matrigel (BD Biosciences #Cat.356231). A 5 µl drop was placed on a slide, gently covered with a coverslip, and then incubated for 20 minutes at RT. After incubation, the slides were imaged under a Nikon Eclipse 80i microscope at 100x magnification, and a time-lapse video of 61 loops for 5 minutes was recorded.

### Generation of the antibodies

Antibodies against Alp5b and P25 were generated in rats. KLH-conjugated peptides of Alp5b (Cys-SNKKKWITKNEYSANP) (GLS #Cat. 998808). and P25 (SPNTQCKNGFLAQMSN-Cys) (GLS #Cat.997976) were synthesized by GL Biochem. The peptides were immunized once with complete Freund’s adjuvant and twice with incomplete Freund’s adjuvant. The rats were bled, and the serum was collected for further analysis.

### In vivo infectivity of sporozoites

Infected mosquitoes were used for sporozoite transmission 22 days post-blood meal, as previously described [74]. Briefly, C57BL/6 mice were anesthetized and kept on the mosquito cages. Fifty mosquitoes per C57BL/6 mouse were used. Infection was observed via Giemsa-stained blood smears.

### In vitro development of exoerythrocytic stages

To observe the development of the parasite in hepatocytes, salivary gland sporozoites were added to HepG2 cells as previously described [75]. Briefly, HepG2 cells were seeded in a 48-well plate (55,000 cells/well) containing collagen-coated 9 mm glass coverslips. The next day, salivary gland sporozoites (5,000/well) were added to the wells, and the plate was spun at 310 × g for 4 minutes. The infected culture was kept in a CO_2_ incubator set at 37°C with 5% CO_2_. The medium was changed every 12 h, and the culture was fixed with 4% paraformaldehyde for 20 minutes at RT.

### Immunofluorescence assay (IFA)

Parasites at different stages were harvested, and IFA was performed as previously described [76]. Briefly, parasites were fixed for 20 minutes at RT with 4% paraformaldehyde. Blood smears and sporozoite slides were permeabilized with 0.1% Triton X-100 (Sigma‒Aldrich #Cat. T8787) for 10 minutes at RT, and liver stage samples were permeabilized with chilled methanol. The samples were blocked with 1% BSA for 1 h at RT and incubated with primary antibodies for 1 h at RT or overnight at 4°C. The primary antibodies used were anti-CSP (diluted 1:1000), anti-HA (diluted 1:1000), anti-actin (diluted 1:1000), anti-g377 (diluted 1:100) [71], anti-tubulin (diluted 1:100), anti-P25 (diluted 1:100), and anti-MSP1 (diluted 1:5,000). The following secondary antibodies were used: Alexa Fluor 488-conjugated anti-rabbit IgG (diluted 1:1,000), (Invitrogen #Cat. A11008), Alexa Fluor 594-conjugated anti-rabbit IgG (diluted 1:1,000), (Invitrogen #Cat. A21442), Alexa Fluor 594-conjugated anti-rat IgG (diluted 1:1,000), (Invitrogen #Cat. A11007), Alexa Fluor 488-conjugated anti-rat IgG (diluted 1:1,000), (Invitrogen #Cat. A11006), Alexa Fluor 488-conjugated anti-mouse IgG (diluted 1:1,000), (Invitrogen #Cat. A11001) and Alexa Fluor 594-conjugated anti-mouse IgG (diluted 1:1,000), (Invitrogen #Cat. A11032). Nuclei were stained with Hoechst 33342 (Invitrogen #Cat. 62249), and samples were mounted with Prolong Diamond antifade reagent (Invitrogen #Cat. P36970). Images were acquired using a confocal laser scanning microscope (Olympus BX61WI) with FV1000 software and a UPlanSAPO 100 × /1.4 NA oil-immersion objective, a widefield fluorescence microscope (Nikon Eclipse 80i) with a Plan Fluor 100 × /1.30 NA oil objective, or a Leica DM3000 LED microscope with a 100 × /1.25 NA oil-immersion objective.

### Analysis of DNA content

Developing male gametocytes were identified by strong anti-tubulin antibody staining, while female gametocytes were distinguished by their diffuse staining pattern. The zygote and ookinetes were immunostained with an anti-P25 antibody. For DNA content analysis of oocysts, mosquito midguts were collected at 8, 12, 15, and 16 days post-feeding, fixed in 4% paraformaldehyde for 30 minutes at room temperature, then washed with cold PBS. The samples were subsequently permeabilized and immunostained with an anti-CSP antibody. Nuclei were stained with Hoechst 33342 and mounted using ProLong Diamond antifade reagent. Fluorescence images were acquired and analyzed using Fiji software (ImageJ). Hoechst-stained DNA fluorescence intensity was quantified by measuring the total fluorescence within each nucleus after subtracting the background signal. The resulting fluorescence intensity values were normalized to those of female gametocytes or single-nucleated parasites. The software (https://theolb.readthedocs.io/en/latest/imaging/measuring-cell-fluorescence-using-imagej.html) was used to measure the fluorescence intensity.

### Determination of the G-actin/F-actin ratio

The G-actin/F-actin ratio was determined via a G-actin/F-actin in vivo assay kit (Cytoskeleton, Inc. #Cat. BK037) according to the manufacturer’s instructions. Blood samples were lysed with saponin, and parasite pellets were washed washed four times with 1X PBS, resuspended in LAS2 buffer, and incubated at 37°C for 10 minutes. Following sequential centrifugation steps—first at 20,913 × g for 5 minutes and then ultracentrifugation at 100,000 × g for 1 hour—the supernatant (G-actin) and pellet (F-actin) fractions were separated. Both fractions were lysed in Laemmli buffer, subjected to 10% SDS-PAGE, and analyzed by western blotting to quantify actin forms.

### Effects of inhibitors on erythrocytic schizogony

Synchronized *P. falciparum* parasites were incubated with different concentrations of cytochalasin-D (Sigma-Aldrich #Cat. C8273), jasplankinolide (Sigma-Aldrich #Cat. 420127), and the Arp2/3 complex inhibitor, CK-666 (Sigma-Aldrich #Cat. 182515) throughout the culture. A schizont culture was set up as described above to examine the effects of the inhibitors on the asexual blood stages of *P. berghei*. The inhibitors were added to the culture and incubated at 37°C for 22–24 h. The growth and morphology of the parasites were monitored via Giemsa-stained smears.

### Immunoprecipitation

Purified gametocytes of the Alp5a-3XHA and WT strains were washed four times with DPBS and crosslinked with 5 mM DSP (dithiobis succinimidyl propionate; Thermo Scientific #Cat. 22586). The DSP-crosslinked samples were incubated for 30 minutes in the dark and centrifuged at 20,913 × g for 5 minutes. The pellet was lysed on ice in 500 µl of RIPA buffer for 30 minutes. The mixture was centrifuged at 20,913 × g for 15 minutes, and the supernatant was transferred to a fresh microcentrifuge tube. A total of 25 µl of anti-HA magnetic beads (Thermo Scientific #Cat. 88838X) was washed with 0.05% TBST four times in a magnetic stand (Stem Cell Technologies #Cat. 18000). The beads were incubated with parasite lysate for 30 min at RT on a tube rotator. After incubation, the beads were washed with TBST followed by ultrapure water, eluted in 2x Laemmli buffer (Bio-Rad #Cat. 1610737), and boiled for 5 minutes. The samples were resolved via 10% SDS‒PAGE, transferred to nitrocellulose membranes, and probed with anti-Alp5b and anti-HA antibodies.

### Western blotting

Western blotting was performed as previously described [76]. Briefly, samples were resolved via 10% SDS‒PAGE and transferred onto a nitrocellulose membrane (Bio-Rad #Cat. 1620112). The membrane was blocked with 1% BSA/PBS and incubated with anti-HA (diluted 1:1,000), (CST #Cat. C29F4) or anti-Hsp70 (diluted 1:1,000) antibodies. The blot was washed 3–4 times with 1X PBST and then incubated with HRP-conjugated anti-rabbit (diluted 1:5,000) (Amersham Biosciences #Cat. NA934V) or anti-mouse IgG (diluted 1:5,000) (Amersham Biosciences #Cat. NA931V). The signals were detected via an enhanced chemiluminescence (ECL) substrate (Bio-Rad, #Cat. 170--5060) and imaged via a ChemiDoc XRS+ system (Bio-Rad, USA).

### Software and statistics

All statistical analyses were conducted using GraphPad Prism 9 software. Comparisons involving more than two groups were performed using one-way ANOVA, while comparisons between two groups were made using Student’s t-test. Statistical significance is indicated as follows: *P < 0.05, **P < 0.01, ***P < 0.001, ****P < 0.0001; n.s., not significant.

## Supporting information

S1 Fig In silico amino acid analysis of ALP5a and ALP5b. (A) The % sequence similarity matrix of Alp5a with *Plasmodium* and other model organisms. (B) The % sequence similarity matrix of Alp5b. The full initials are as follows: *Pb- Plasmodium berghei, Py- Plasmodium yoelii, Pm- Plasmodium malariae, Pv- Plasmodium vivax, Pk- Plasmodium knowlesi, Pcy- Plasmodium cynomolgi, Hc- Hepatocystis, Tg- Toxoplasma gondii, Pf- Plasmodium falciparum, Sc-Saccharomyces cerevisiae, Gm- Glycine max, Dm- Drosophila melanogaster, At- Arabidopsis thaliana, Ce- Caenorhabditis elegans, Mm- Mus musculus, Hs- Homo sapiens* and *Dd- Dictyostelium discoideum, Ld- Leishmania donovani* and *Dn- Danio.*
(TIF)

S2 Fig Protein–protein interaction networks of Alp5s in *P. berghei* identified via STRING analysis. (A) Network diagram depicting protein–protein interactions involving Alp5a. The network highlights direct and indirect interactions, illustrating the connectivity and potential functional relationships among Alp5a and associated proteins. (B) Table listing proteins identified to interact with Alp5a. (C) Protein association network for Alp5b, showing substantial overlap with the Alp5a interactome, suggesting shared functional pathways. (D) Table listing proteins identified to interact with Alp5b.
(TIF)

S3 Fig Structural superposition of PbAlp5a and Arp3. PbAlp5a (green) and Arp3 (pink) were superimposed based on the conserved phenylalanine residues (Phe425 of PbAlp5a, atom 2249; and Phe282 of Arp3, atom 6856). The alignment yields a root-mean-square deviation (RMSD) of 2.961 Å, indicating structural similarity at the alignment site.
(EPS)

S4 Fig To investigate potential protein–protein interactions, ClusPro docking simulations were performed to predict complex formation, and binding energy calculations were used to assess interaction strength. The results indicate that Alp5a exhibits a higher binding affinity for both actin isoforms compared to Alp5b. The binding interactions of Alp5 isoforms with actin variants reveal distinct affinities. Alp5a exhibits stronger interactions, with binding energies of -1187.1 kcal/mol for the Alp5a–actin 1 complex and -1369.1 kcal/mol for the Alp5a–actin 2 complex, indicating a more favorable binding, particularly with actin 2. In contrast, Alp5b shows comparatively weaker binding, with energies of -679.8 kcal/mol (Alp5b–actin 1) and -844.8 kcal/mol (Alp5b–actin 2). (Alp5a-Green, Alp5b-magenta, Actin 1-wheatish, Actin 2-olive orange). **(A)** Cartoon structure of *P. berghei* Actin 1. **(B)** Interaction between *P. berghei* Actin 1 and Alp5a. (**C)** Interaction between *Plasmodium berghei* Actin 1 and Alp5b. **(D)** Cartoon structure of *P. berghei* Actin 2. **(E)** Interaction between *P. berghei* Actin 2 and Alp5a. (**F)** Interaction between *P. berghei* Actin 2 and Alp5b.
(TIF)

S5 Fig Structural superposition of AlphaFold2-predicted and experimentally determined crystal structures of *Plasmodium* actins. (A) *P. falciparum* actin I: AlphaFold2 model (wheat) overlaid with the crystal structure (PDB ID: 6I4H; pink). Root-mean-square deviation (RMSD) = 0.627 Å. (B) *P. berghei* actin II: AlphaFold2 model (olive) overlaid with the crystal structure (PDB ID: 6I4M; salmon red); RMSD = 0.796 Å.
(EPS)

S6 Fig Interaction comparison of the Arp2–Arp3 and the PbAlp5a–PbAlp5b. (A) Cartoon representation of the interaction between Arp2 (yellow) and Arp3 (sky blue). (B) Cartoon representation of the interaction between PbAlp5a (green) and PbAlp5b (magenta). (C) Superposition of the PbAlp5a (green)–PbAlp5b (magenta) onto the Arp2 (yellow)–Arp3 (sky blue) reveals structural similarity. PbAlp5a aligns with Arp3 (RMSD = 2.905 Å), and PbAlp5b aligns with Arp2 (RMSD = 1.899 Å), suggesting a conserved interaction interface.
(TIF)

S7 Fig Generation of transgenic parasites of Alp5b and Alp5a. **(A)** To check the expression of Alp5b, the gene was endogenously tagged with 3XHA-mCherry. The arrows and lollipops indicate the 5’ and 3’ UTRs, respectively. (**B**) Correct site-specific 5’ and 3’ integration was confirmed via diagnostic PCR via the primer sets 1724/1392 and 1215/1666, respectively. No bands were amplified from WT genomic DNA. (**C**) Western blot analysis of gametocyte lysates expressing the Alp5b-3XHA-mCherry fusion protein. The fusion protein (~77 kDa) was detected using an anti-HA antibody. The blot was reprobed with an anti-Hsp70 antibody to serve as a loading control. **(D)** To check the expression of Alp5a, the gene was endogenously tagged with 3XHA. **(E)** Correct site-specific 5’ and 3’ integration was confirmed via diagnostic PCR via primers 2130/1218 and 1215/2090, respectively. No bands were amplified from WT genomic DNA. (**F**) Western blot analysis of the Alp5a-3XHA fusion protein (70.5 kDa) via an anti-HA antibody. The blot was reprobed with an anti-Hsp70 antibody as a loading control. The expected size bands were observed in the transgenic parasites, while no bands were detected in the WT lane.
(EPS)

S8 Fig Alp5a and Alp5b were not expressed in the sporozoite or liver stages. (A) Confocal immunofluorescence analysis of Alp5a and Alp5b in -3XHA tagged transgenic parasites in sporozoites. Parasites were immunostained with anti-HA and anti-CSP antibodies. (B) Confocal immunofluorescence analysis of Alp5a and Alp5b expression in exoerythrocytic forms (EEFs). Infected hepatocytes were immunostained with anti-HA and anti-MSP1 antibodies. No HA signal was detected in either stage, indicating the absence of Alp5a and Alp5b expression.
(EPS)

S9 Fig Generation of Alp5 KO and complemented parasites. **(A)** The Alp5b gene was disrupted by double-crossover (DCO) homologous recombination with fragments F1 and F2, as shown in the targeting cassette. The targeting vector pBC-GFP-hDHFR:yFCU consists of the GFP and hDHFR:yFCU cassettes regulated by 5’ (arrow) and 3’ (lollipop) regulatory sequences. **(B)** Diagnostic PCR showing the correct 5’ and 3’ integrations via the primer sets 1665/1225 and 1215/1666, respectively. The primers 1671/1672 were used to confirm the absence of the Alp5b locus in the KO parasites, and the band was amplified in the WT parasites but not in the KO parasites. **(C)** Schematic representation of the complementation strategy for reintroducing the Alp5b gene at the KO locus. After being transfected with the Alp5b-targeting cassette, the parasites were negatively selected with the 5-FC drug. **(D)** Primers 1671/1672 amplified the target locus in WT and complemented lines, but no amplification was detected in KO parasites. **(E)** Similar to Alp5b, Alp5a was disrupted by DCO. The targeting vector pBC-mCherry-tgDHFR consists of the mCherry and tgDHFR cassettes regulated by 5’ (arrow) and 3’ (lollipop) regulatory sequences. **(F)** Correct site-specific 5’ and 3’ integrations were confirmed via the primer sets 2089/1225 and 1913/2090, respectively. The absence of a locus in the KO line was confirmed via the primers 2091/2092. **(G)** Schematic showing the Alp5a gene complementation strategy. The Alp5a expression cassette was cloned into the pBC-P230-hDHFR plasmid, linearized, and transfected into Alp5a KO schizonts, which were subsequently selected via the WR drug. **(H)** Site-specific 5’ and 3’ integrations of the targeting cassette at the P230 locus were confirmed via diagnostic PCR via primers 2270/2271 and 1215/2272, respectively. **(I)** Amplification of the Alp5a ORF in the complemented line via the primer set 2091/2092.
(EPS)

S10 Fig Attempts to generate Alp5a/Alp5b double-KO parasites. **(A)** Schematic representation of the strategy for generating Alp5a*/*Alp5b double-KO parasites. *Pb*Alp5a KO schizonts were transfected with an Alp5b-targeting cassette. (a) ALP5a locus (b) Recombination at the Alp5b locus. (c) Expected double KO locus. **(B)** Schematic representation of the second strategy. Both targeting constructs were transfected simultaneously into *P. berghei* schizonts.
(TIF)

S11 Fig Effect of inhibitors on the growth of *P. falciparum* and *P. berghei* blood stage parasites. **(A)** Counting of ring, trophozoite, and schizont stages in *P. falciparum* cultures after treatment. There was no significant difference in the number of schizonts between the cytochalasin D-treated group and the DMSO-treated group at 24 h (P = 0.9488; one-way ANOVA). The number of schizonts significantly decreased in the jasplankinolide (****P < 0.0001; one-way ANOVA)- and CK-666 (***P = 0.003; unpaired Student’s t test)-treated groups. Cytochalasin D and jasplankinolide did not affect the ring stage at 24 h (P = 0.0638; one-way ANOVA). The number of ring stages significantly decreased in the CK-666-treated groups (***P = 0.0008; one-way ANOVA). There were fewer schizont numbers at 48 h in all the treated groups (****P < 0.0001; one-way ANOVA). The number of ring stages observed at 48 h also decreased significantly (****P < 0.0001; one-way ANOVA). The number of trophozoites was significantly lower in all the treated groups (***P = 0.0001; one-way ANOVA). All the stages observed at 72 h were significantly reduced in all the treated groups (****P < 0.0001; one-way ANOVA). Data are presented as the mean ± SEM from three independent biological replicates. **(B)** *P. berghei* blood cultures were treated with the inhibitors cytochalasin D (1 µM), jasplankinolide (250 nM) and CK-666 (300 µM). Parasitemia was comparable between the treated and control groups (P = 0.9984; Brown-Forsythe ANOVA). Data are presented as the mean ± SEM from three independent biological replicates.
(TIF)

S12 Fig Alp5s are essential for oocyst development and malaria transmission. (A) Representative widefield fluorescence microscopy images of mosquito midguts showing oocysts on the indicated days post-infection. **(B)** Quantification of oocyst numbers. There was a significant difference in oocyst number between WT and KO parasites on days 8–18 (****P < 0.0001; Kruskal‒Wallis test). In contrast, no differences were detected between the complement lines and WT on days 8 (P = 0.8721), 10 (P = 0.5445), 12 (P = 0.0662), 14 (P = 0.1075), 16 (P = 0.7252) or 18 (P = 0.8941). Sixty midguts from all the groups were dissected each day. **(C)** Determination of the oocyst area. The data from independent clones were pooled, and a significant difference was detected between the WT and KO lines on days 8–18 (****P < 0.0001), whereas no differences were detected between the complemented lines and the WT line on days 8 (P = 0.1058), 10 (P = 0.0890), (P = 0.1553), 14 (P = 0.8983), 16 (P = 0.5481) or 18 (P = 0.5198). The Kruskal‒Wallis test was used to determine the significance. Two hundred oocyst areas in the WT, Alp5a KO, and Alp5b KO lines and 40 oocyst areas in the complement lines were compared on the indicated days. The triangle represents the mean individual count in each experiment, and each dot represents an individual count. Data are presented as the mean ± SEM from three independent biological replicates.
(TIF)

S13 Fig Hypoploidy of microgametes does not affect the viability of ookinetes. **(A)** Widefield fluorescence microscopy revealed that the morphology of Alp5 KO ookinetes was comparable to that of WT parasites. **(B)** There was no difference in the number of ookinetes between WT and Alp5 KO parasites (P = 0.9275, one-way ANOVA). Ookinete numbers from 109 (WT-mCh), 72 (Alp5b cl1), 75 (Alp5b cl2), 110 (Alp5b comp1), 65 (Alp5b cl2), 75 (Alp5a cl1), 105 (Alp5a cl2), 65 (Alp5a comp1), and 85 (Alp5a comp2) midguts were observed. **(C)** Ookinetes were allowed to glide for 5 minutes with 61 loops using Matrigel. No difference in gliding was observed between WT and Alp5 KO parasites.
(EPS)

S14 Fig Generation of male and female gamete-defective parasite lines. **(A)** Schematic representation of the strategy used to generate the male gamete-defective Pb48/45 KO parasite line. **(B)** Genotyping confirmed correct site-specific integration using primers 2229/2036. The Pb48/45 open reading frame (ORF) was amplified from WT genomic DNA but not from Pb48/45 KO parasites using primers 2230/2231. **(C)** Strategy used to generate the female gamete-defective Pb47 KO parasite line. (D) Site-specific integration was verified by PCR using primers 2232/2035. The Pb47 ORF was amplified from WT genomic DNA but not from Pb47 KO parasites using primers 2233/2234.
(EPS)

S15 Fig Summary of the genetic cross experiments. Genetic crosses involving the KO line and either WT parasites or female gamete-defective mutants successfully restored the KO phenotype. In contrast, no phenotype rescue was observed when the KO line was crossed with a male gamete-defective mutant.
(EPS)

S16 Fig Infected salivary glands showing the sporozoite load. **(A)** Sporozoite loads in the salivary glands were assessed using widefield fluorescence microscopy following genetic crosses between WT-mCherry and Alp5b KO, and between WT-GFP and Alp5a KO parasite lines. **(B)** The salivary gland sporozoite load in Alp5 KO lines after genetic crosses with male (Pb48/45 KO) or female (Pb47 KO) gamete-defective lines.
(EPS)

S17 Fig In vivo and in vitro infectivity of sporozoites. **(A)** Salivary gland sporozoites were injected into C57BL/6 mice, and the pre-patent period was observed. Genetic crosses with WT or female gamete-defective Pb47 KO lines restored the infection of Alp5 KO parasites, n = 5 number of mice per group. **(B and C).** The Alp5 ORF was amplified in the KO lines after genetic crosses with WT or gamete-defective lines. **(D)** Sporozoites invade hepatocytes and develop into EEFs. **(E)** Confocal immunofluorescence analysis of EEF at 65 h. Only GFP- or mCherry-expressing EEFs were counted (P = 0.0635; Kruskal‒Wallis test).
(EPS)

S1 Table List of primers used in this study. (DOCX)

S2 Table Protein-protein interaction prediction score. (DOCX)

S1 Data Raw data supporting all the quantitative analyses used to generate graphs in the manuscript. Excel sheet comprises data used to generate both main and supplementary figures. Each sheet is labelled according to corresponding figures and panels.
(XLSX)

S1 File Raw images. **Uncropped images of all the western blots and gels used in main and supplementary figures.** Blots and gels are labelled with respective figures and panels.
(DOCX)
