# Supplementary figures and images for: Plasmodium actin-like proteins are essential for DNA segregation during male gametogenesis and malaria transmission
Source: PLoS Pathog. 2025 Nov 11;21(11):e1013687. doi: 10.1371/journal.ppat.1013687 (PMC12617974; doi:10.1371/journal.ppat.1013687)

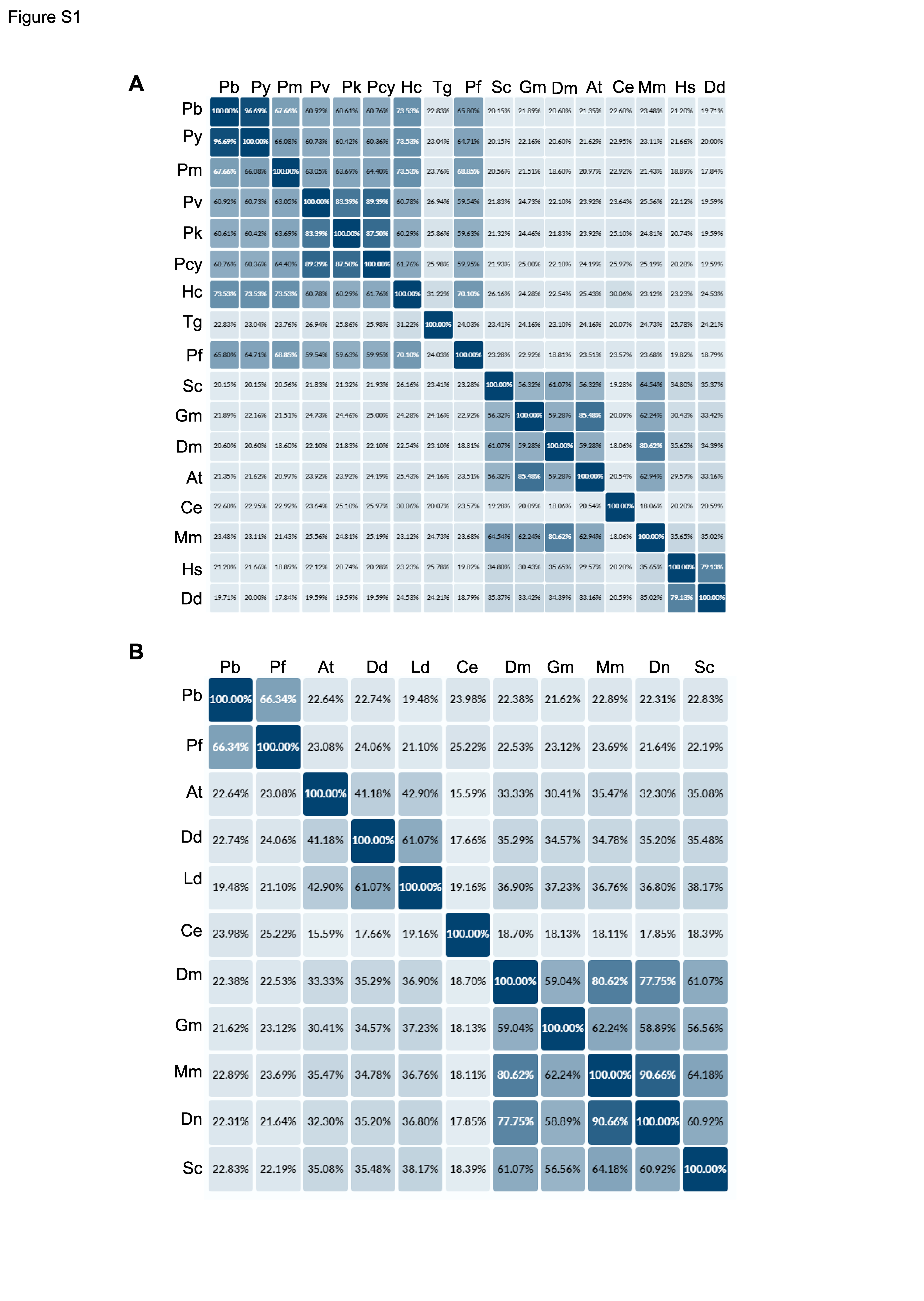

Supplement: S1 Fig — (A) The % sequence similarity matrix of Alp5a with Plasmodium and other model organisms. (B) The % sequence similarity matrix of Alp5b. The full initials are as follows: Pb- Plasmodium berghei, Py- Plasmodium yoelii, Pm- Plasmodium malariae, Pv- Plasmodium vivax, Pk- Plasmodium knowlesi, Pcy- Plasmodium cynomolgi, Hc- Hepatocystis, Tg- Toxoplasma gondii, Pf- Plasmodium falciparum, Sc-Saccharomyces cerevisiae, Gm- Glycine max, Dm- Drosophila melanogaster, At- Arabidopsis thaliana, Ce- Caenorhabditis elegans, Mm- Mus musculus, Hs- Homo sapiens and Dd- Dictyostelium discoideum, Ld- Leishmania donovani and Dn- Danio. (TIF) [file ppat.1013687.s001.tif]

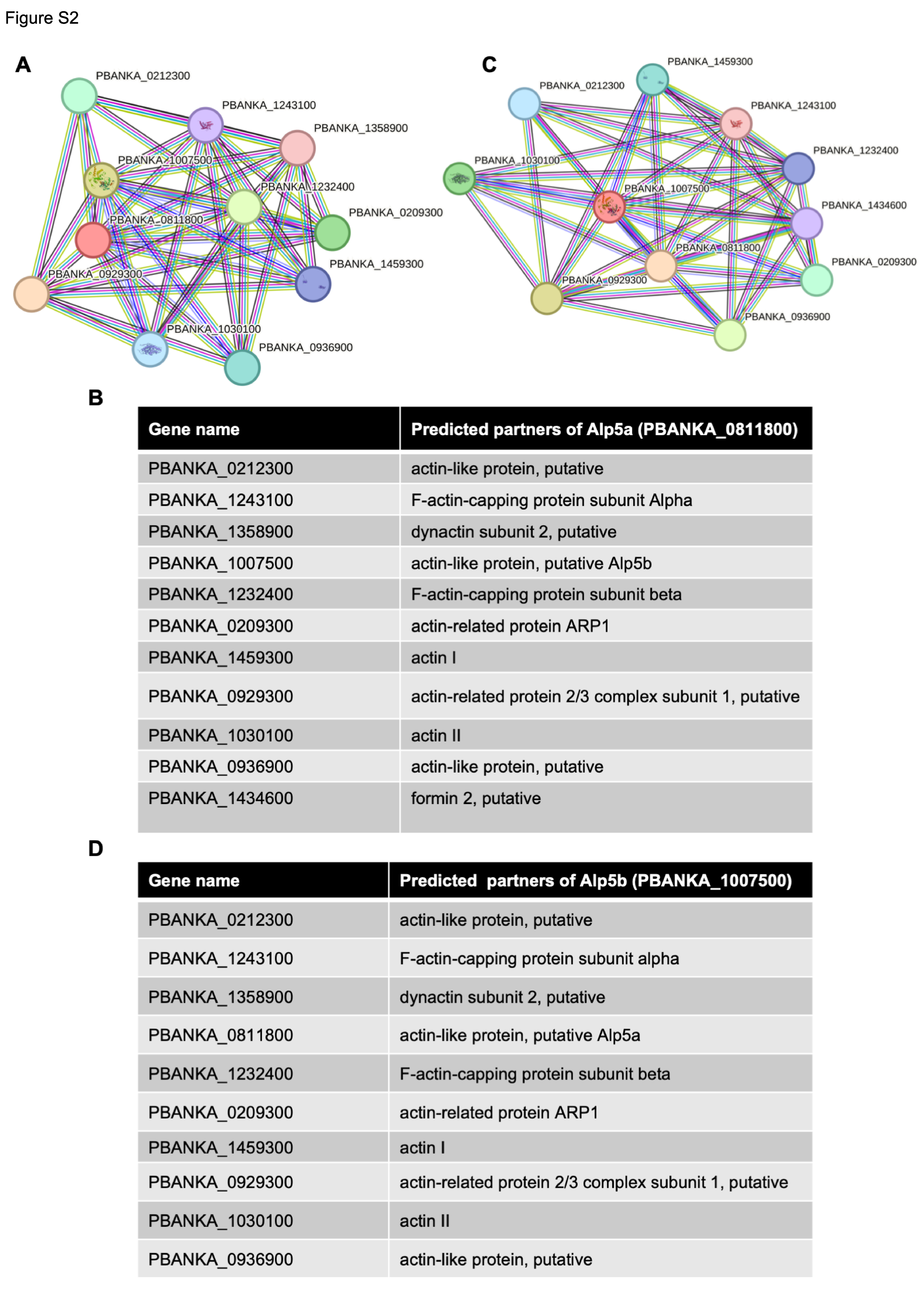

Supplement: S2 Fig — (A) Network diagram depicting protein–protein interactions involving Alp5a. The network highlights direct and indirect interactions, illustrating the connectivity and potential functional relationships among Alp5a and associated proteins. (B) Table listing proteins identified to interact with Alp5a. (C) Protein association network for Alp5b, showing substantial overlap with the Alp5a interactome, suggesting shared functional pathways. (D) Table listing proteins identified to interact with Alp5b. (TIF) [file ppat.1013687.s002.tif]

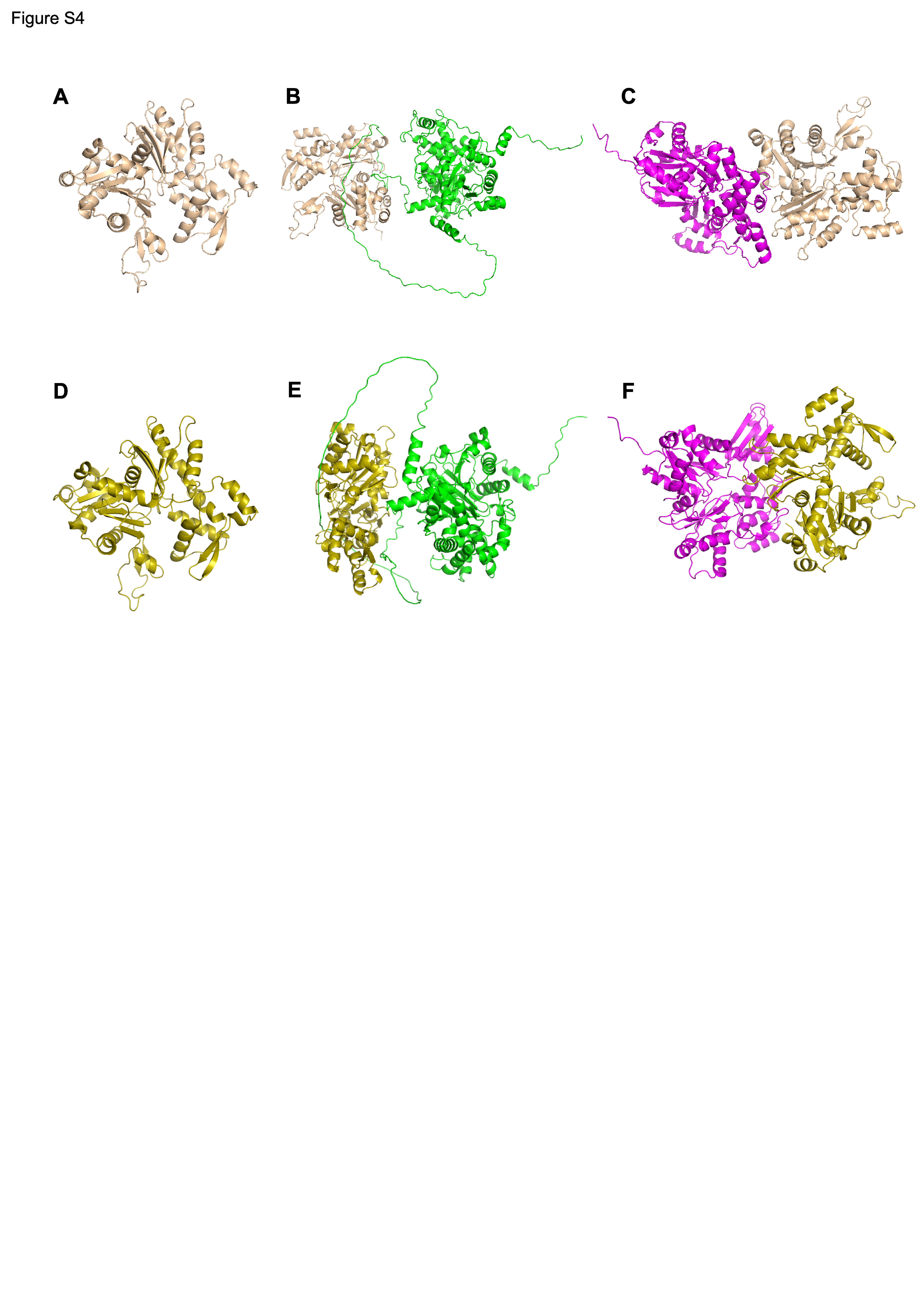

Supplement: S4 Fig — The results indicate that Alp5a exhibits a higher binding affinity for both actin isoforms compared to Alp5b. The binding interactions of Alp5 isoforms with actin variants reveal distinct affinities. Alp5a exhibits stronger interactions, with binding energies of -1187.1 kcal/mol for the Alp5a–actin 1 complex and -1369.1 kcal/mol for the Alp5a–actin 2 complex, indicating a more favorable binding, particularly with actin 2. In contrast, Alp5b shows comparatively weaker binding, with energies of -679.8 kcal/mol (Alp5b–actin 1) and -844.8 kcal/mol (Alp5b–actin 2). (Alp5a-Green, Alp5b-magenta, Actin 1-wheatish, Actin 2-olive orange). (A) Cartoon structure of P. berghei Actin 1. (B) Interaction between P. berghei Actin 1 and Alp5a. (C) Interaction between Plasmodium berghei Actin 1 and Alp5b. (D) Cartoon structure of P. berghei Actin 2. (E) Interaction between P. berghei Actin 2 and Alp5a. (F) Interaction between P. berghei Actin 2 and Alp5b. (TIF) [file ppat.1013687.s004.tif]

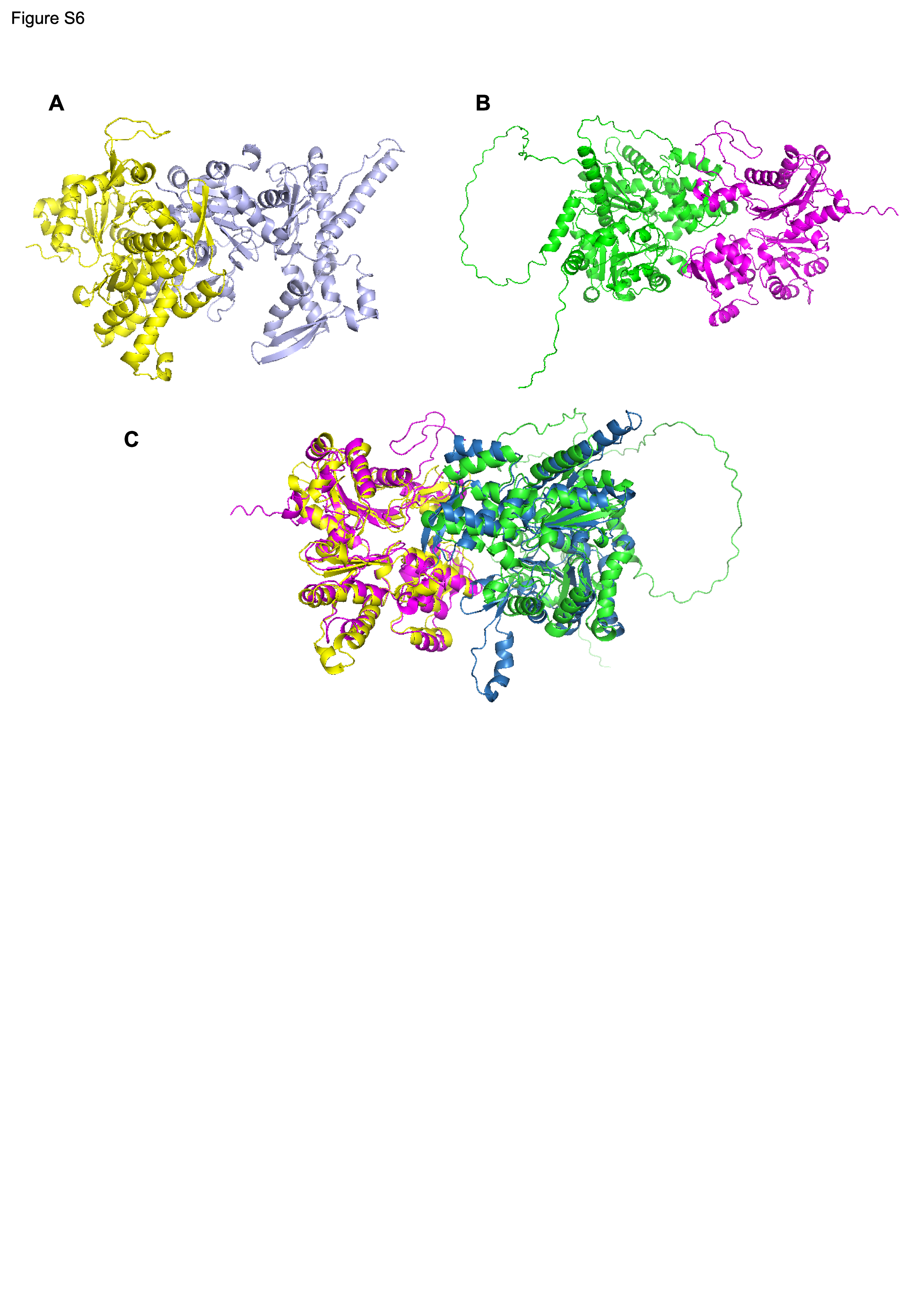

Supplement: S6 Fig — (A) Cartoon representation of the interaction between Arp2 (yellow) and Arp3 (sky blue). (B) Cartoon representation of the interaction between PbAlp5a (green) and PbAlp5b (magenta). (C) Superposition of the PbAlp5a (green)–PbAlp5b (magenta) onto the Arp2 (yellow)–Arp3 (sky blue) reveals structural similarity. PbAlp5a aligns with Arp3 (RMSD = 2.905 Å), and PbAlp5b aligns with Arp2 (RMSD = 1.899 Å), suggesting a conserved interaction interface. (TIF) [file ppat.1013687.s006.tif]

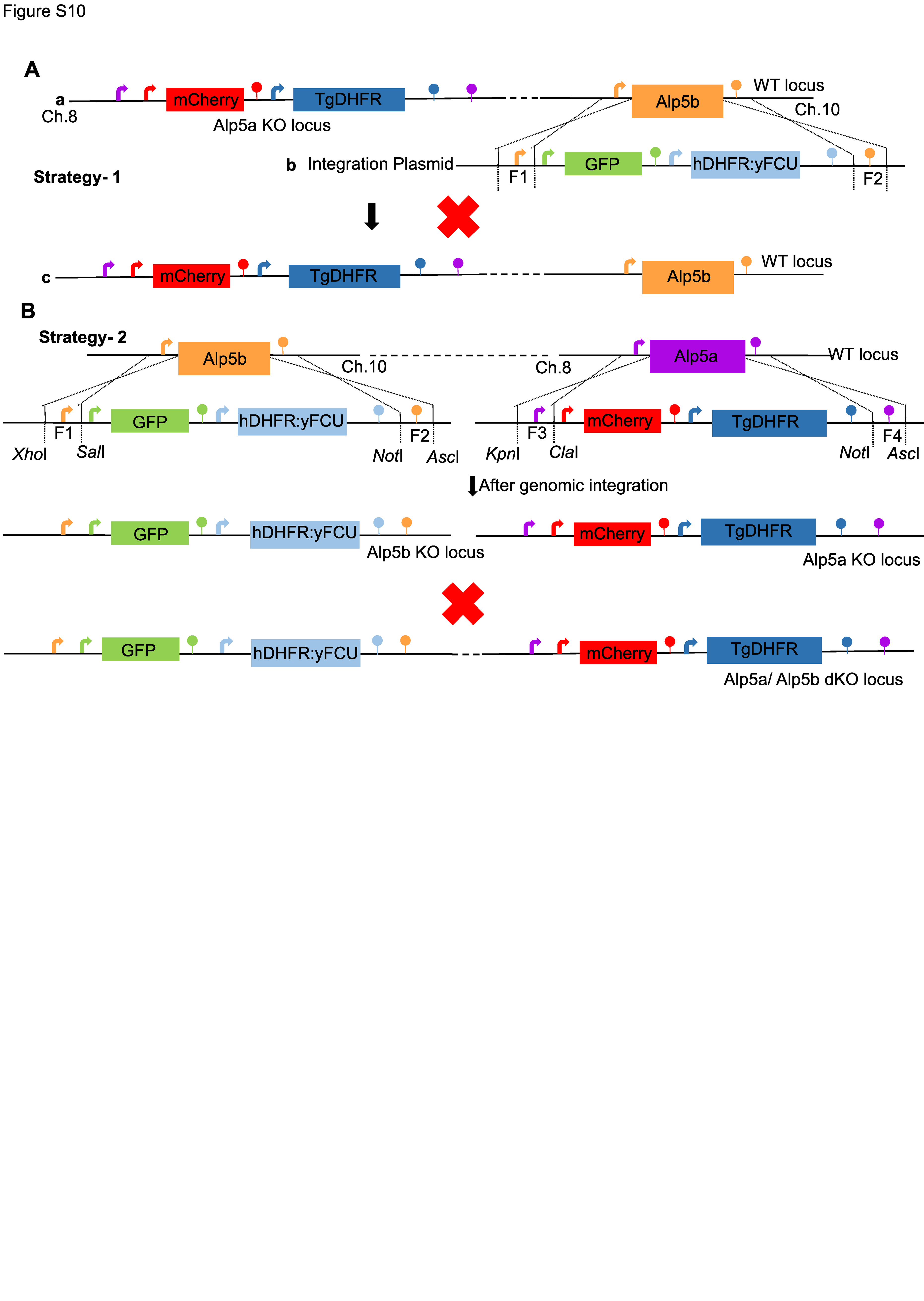

Supplement: S10 Fig — (A) Schematic representation of the strategy for generating Alp5a/Alp5b double-KO parasites. PbAlp5a KO schizonts were transfected with an Alp5b-targeting cassette. (a) ALP5a locus (b) Recombination at the Alp5b locus. (c) Expected double KO locus. (B) Schematic representation of the second strategy. Both targeting constructs were transfected simultaneously into P. berghei schizonts. (TIF) [file ppat.1013687.s010.tif]

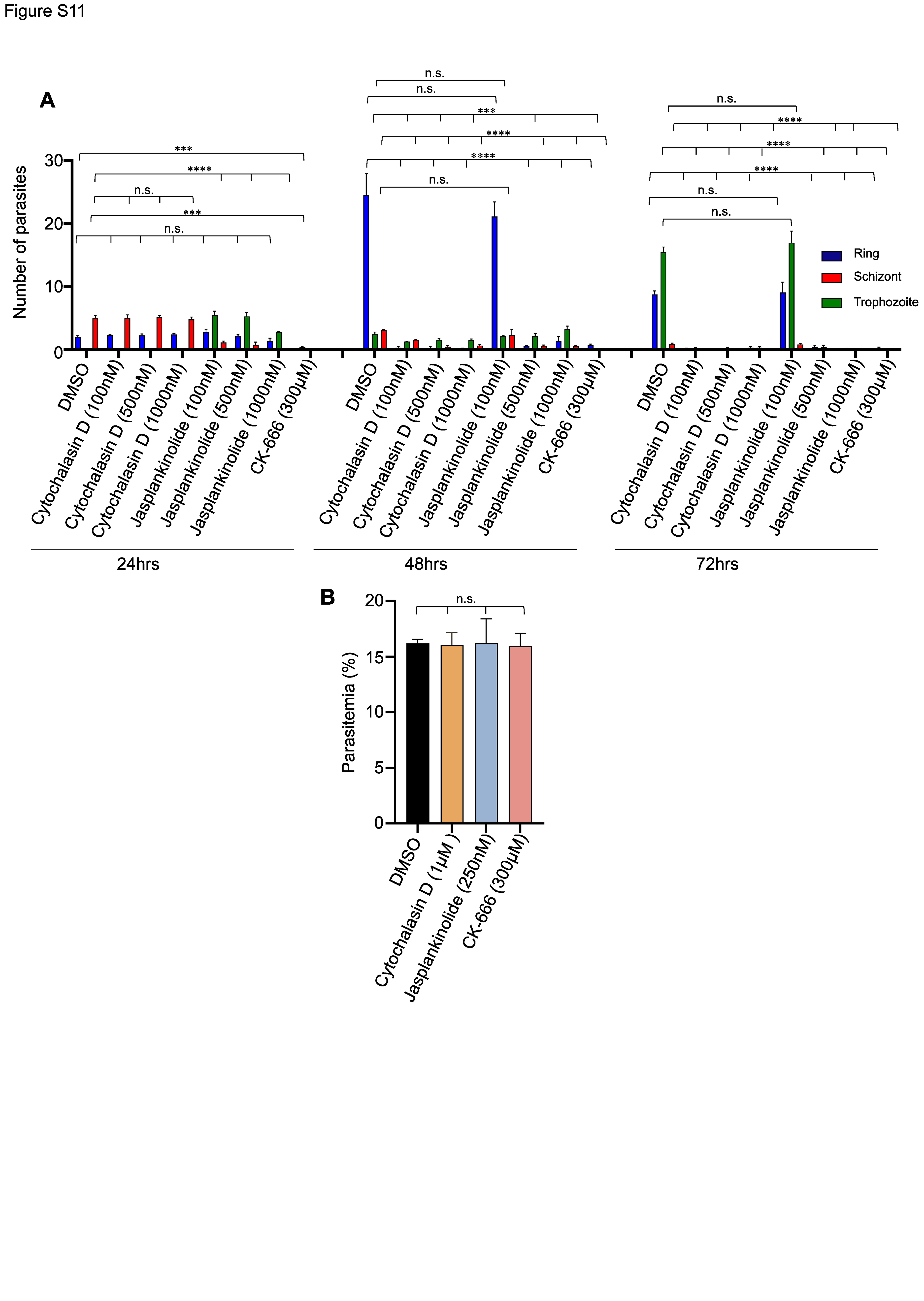

Supplement: S11 Fig — (A) Counting of ring, trophozoite, and schizont stages in P. falciparum cultures after treatment. There was no significant difference in the number of schizonts between the cytochalasin D-treated group and the DMSO-treated group at 24 h (P = 0.9488; one-way ANOVA). The number of schizonts significantly decreased in the jasplankinolide (****P < 0.0001; one-way ANOVA)- and CK-666 (***P = 0.003; unpaired Student’s t test)-treated groups. Cytochalasin D and jasplankinolide did not affect the ring stage at 24 h (P = 0.0638; one-way ANOVA). The number of ring stages significantly decreased in the CK-666-treated groups (***P = 0.0008; one-way ANOVA). There were fewer schizont numbers at 48 h in all the treated groups (****P < 0.0001; one-way ANOVA). The number of ring stages observed at 48 h also decreased significantly (****P < 0.0001; one-way ANOVA). The number of trophozoites was significantly lower in all the treated groups (***P = 0.0001; one-way ANOVA). All the stages observed at 72 h were significantly reduced in all the treated groups (****P < 0.0001; one-way ANOVA). Data are presented as the mean ± SEM from three independent biological replicates. (B) P. berghei blood cultures were treated with the inhibitors cytochalasin D (1 µM), jasplankinolide (250 nM) and CK-666 (300 µM). Parasitemia was comparable between the treated and control groups (P = 0.9984; Brown-Forsythe ANOVA). Data are presented as the mean ± SEM from three independent biological replicates. (TIF) [file ppat.1013687.s011.tif]

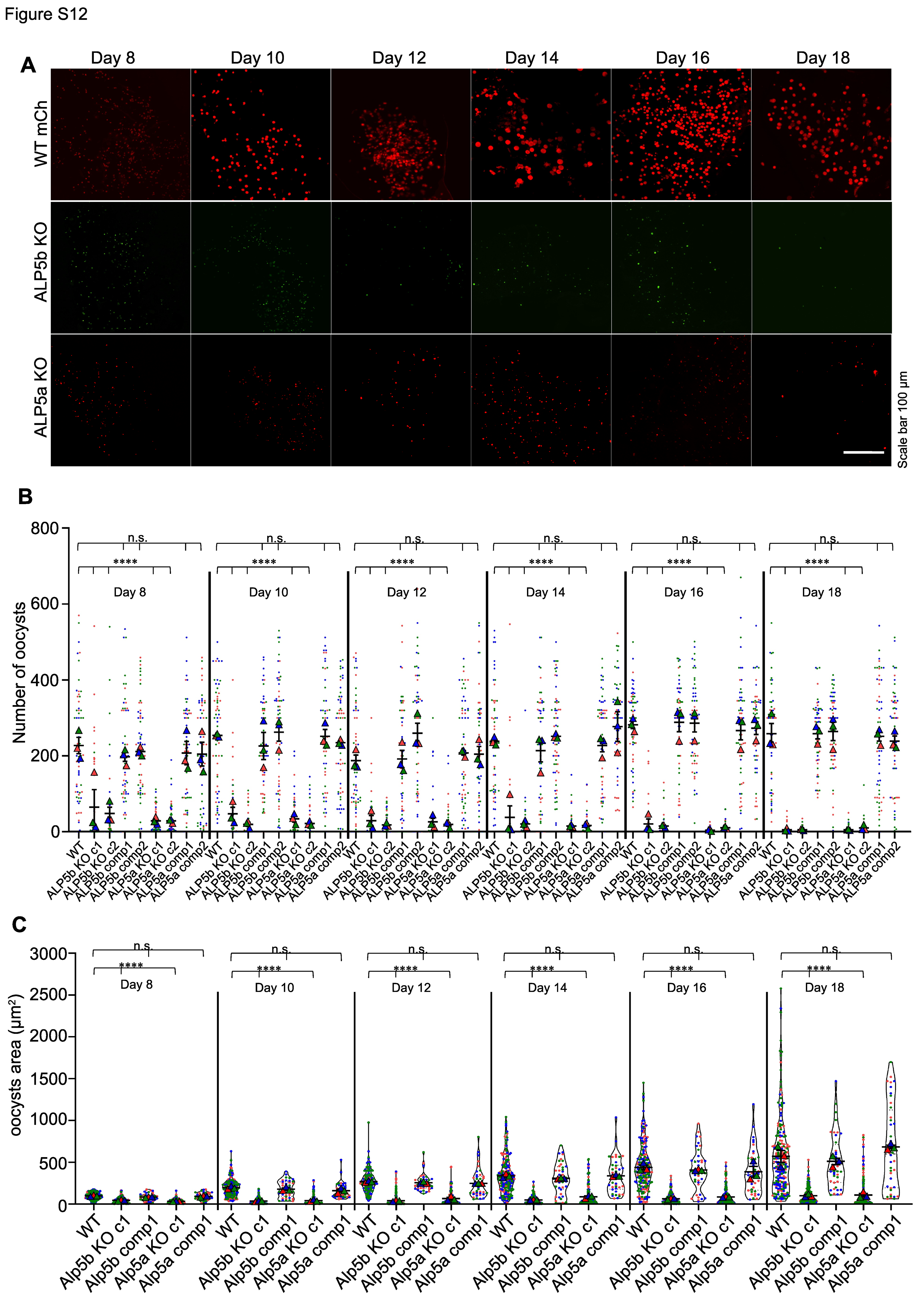

Supplement: S12 Fig — (A) Representative widefield fluorescence microscopy images of mosquito midguts showing oocysts on the indicated days post-infection. (B) Quantification of oocyst numbers. There was a significant difference in oocyst number between WT and KO parasites on days 8–18 (****P < 0.0001; Kruskal‒Wallis test). In contrast, no differences were detected between the complement lines and WT on days 8 (P = 0.8721), 10 (P = 0.5445), 12 (P = 0.0662), 14 (P = 0.1075), 16 (P = 0.7252) or 18 (P = 0.8941). Sixty midguts from all the groups were dissected each day. (C) Determination of the oocyst area. The data from independent clones were pooled, and a significant difference was detected between the WT and KO lines on days 8–18 (****P < 0.0001), whereas no differences were detected between the complemented lines and the WT line on days 8 (P = 0.1058), 10 (P = 0.0890), (P = 0.1553), 14 (P = 0.8983), 16 (P = 0.5481) or 18 (P = 0.5198). The Kruskal‒Wallis test was used to determine the significance. Two hundred oocyst areas in the WT, Alp5a KO, and Alp5b KO lines and 40 oocyst areas in the complement lines were compared on the indicated days. The triangle represents the mean individual count in each experiment, and each dot represents an individual count. Data are presented as the mean ± SEM from three independent biological replicates. (TIF) [file ppat.1013687.s012.tif]

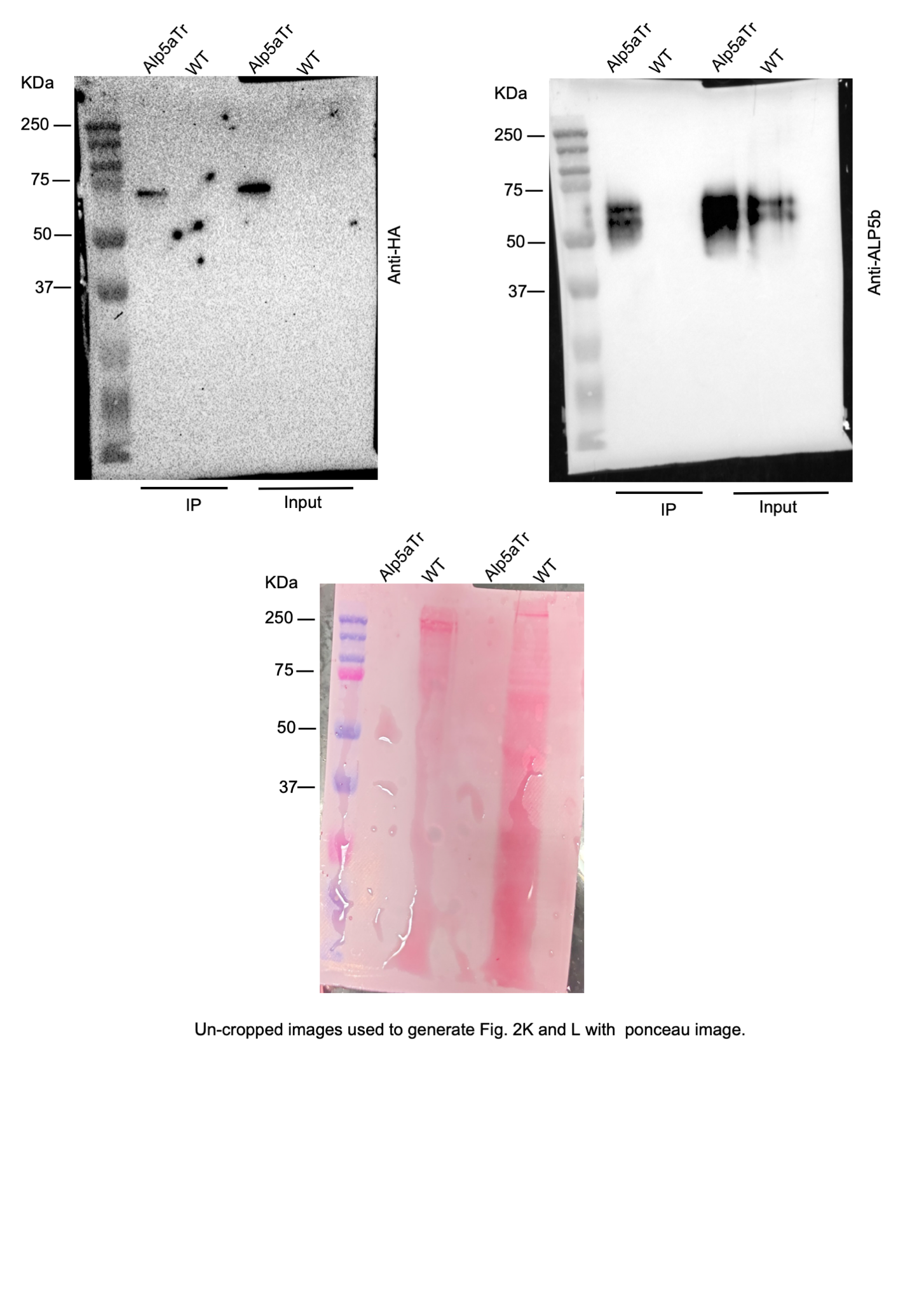


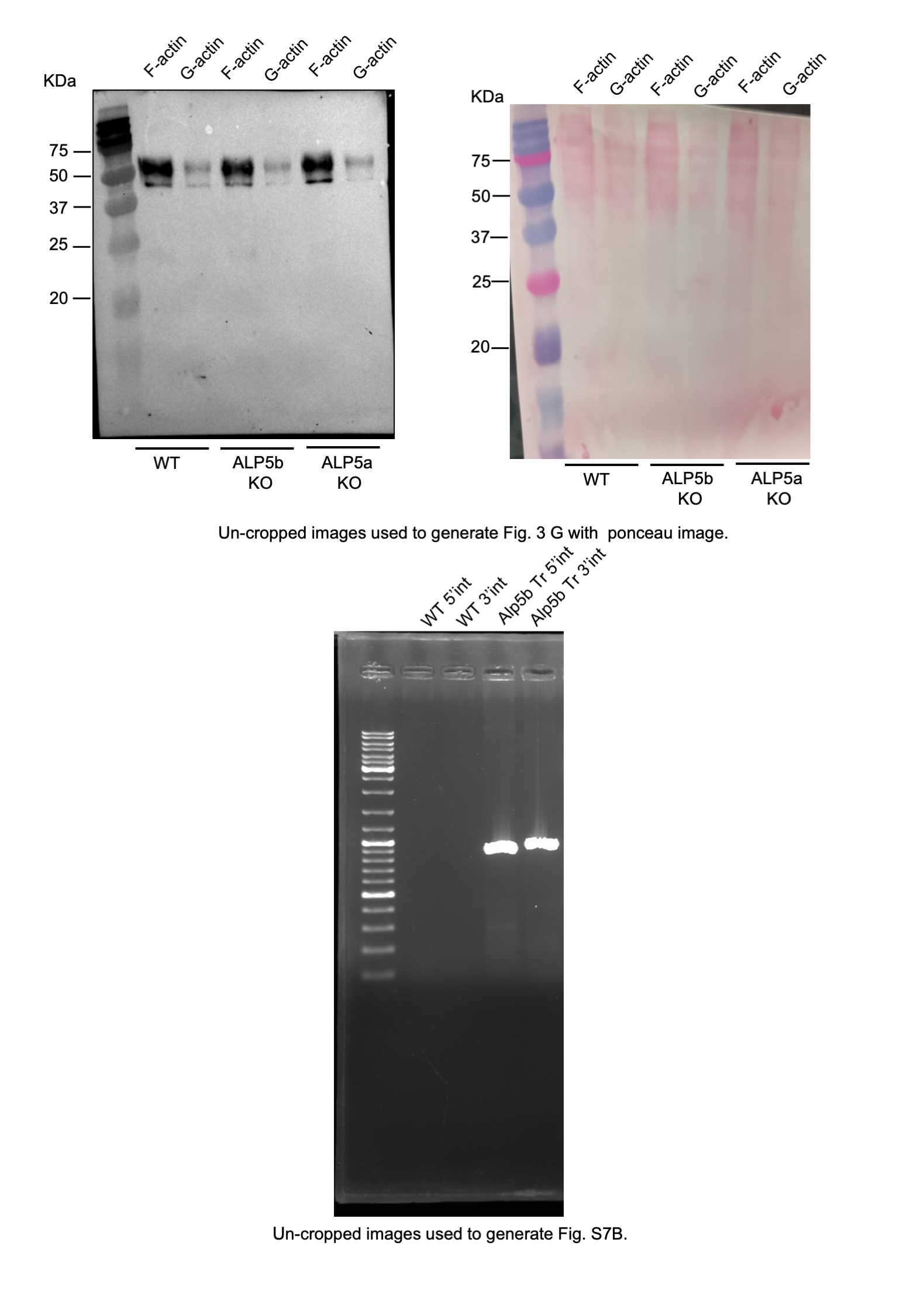


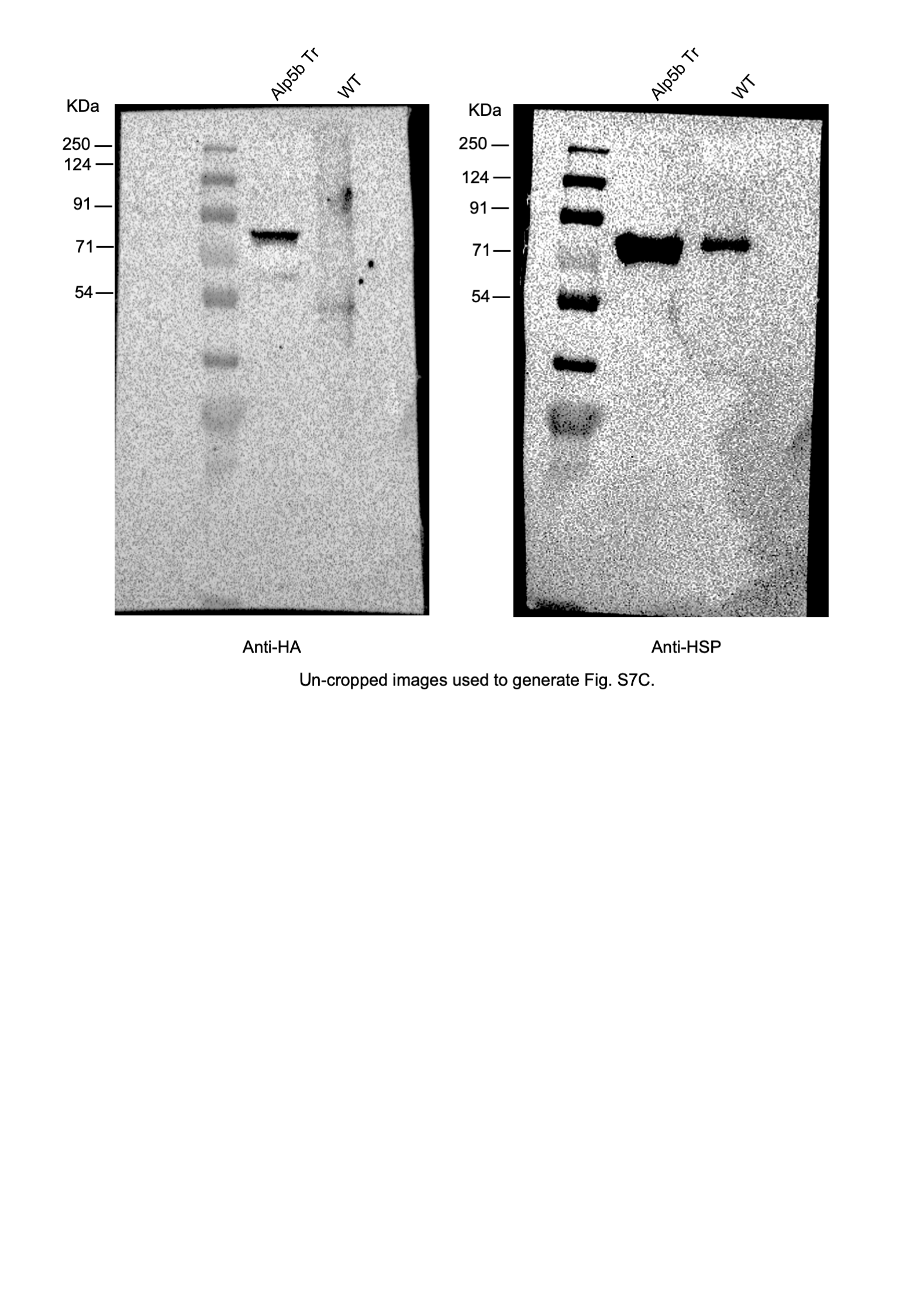


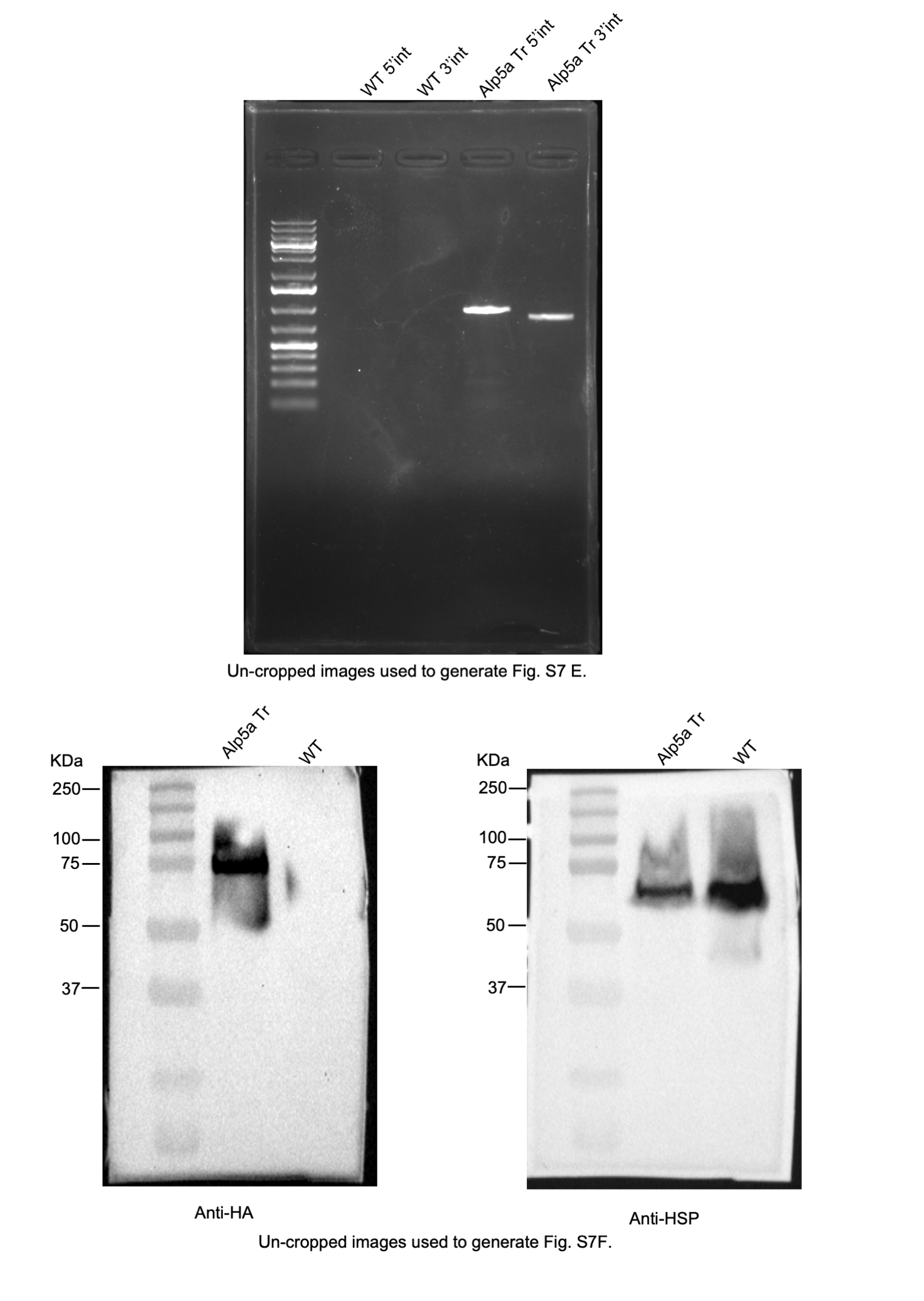


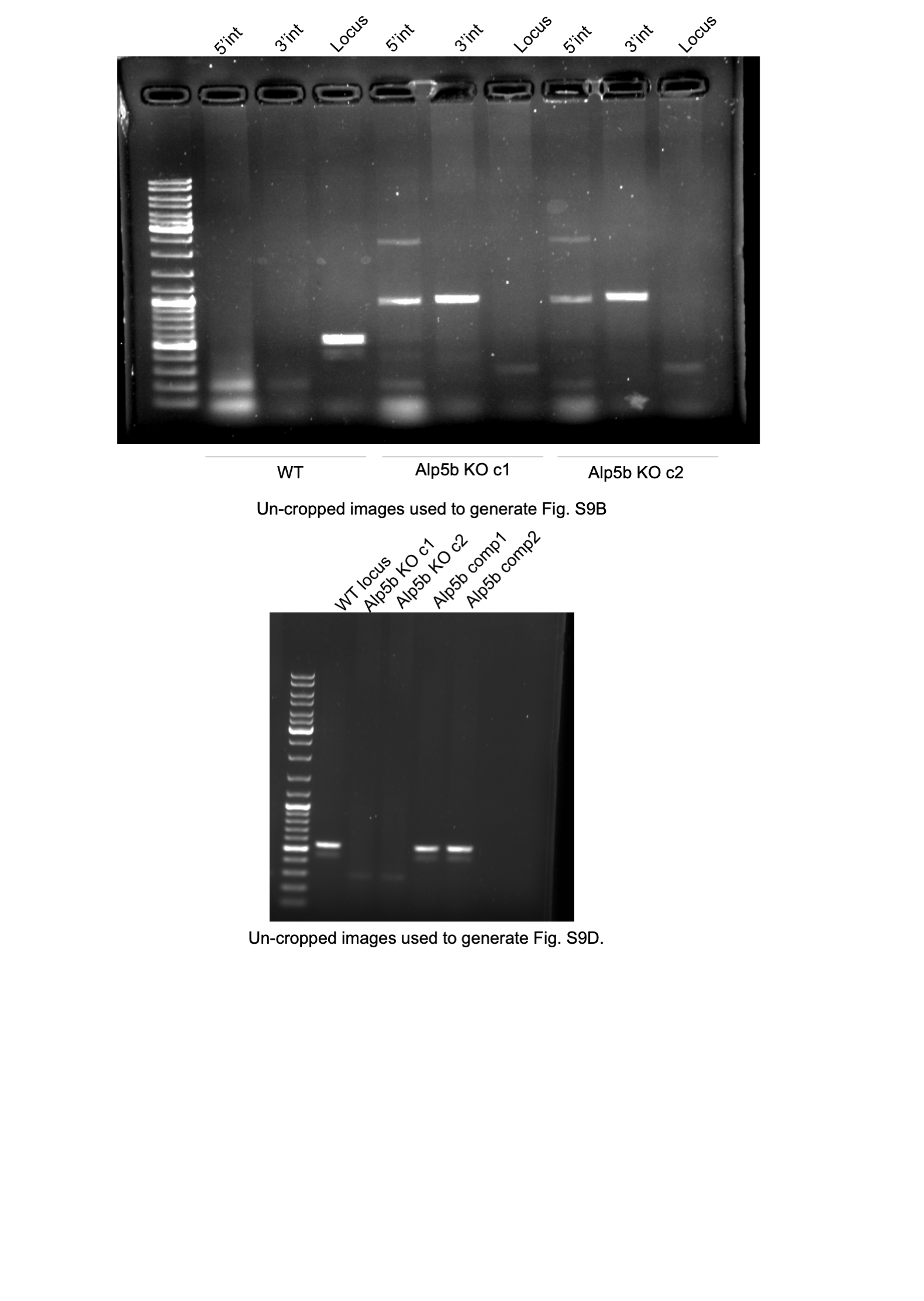


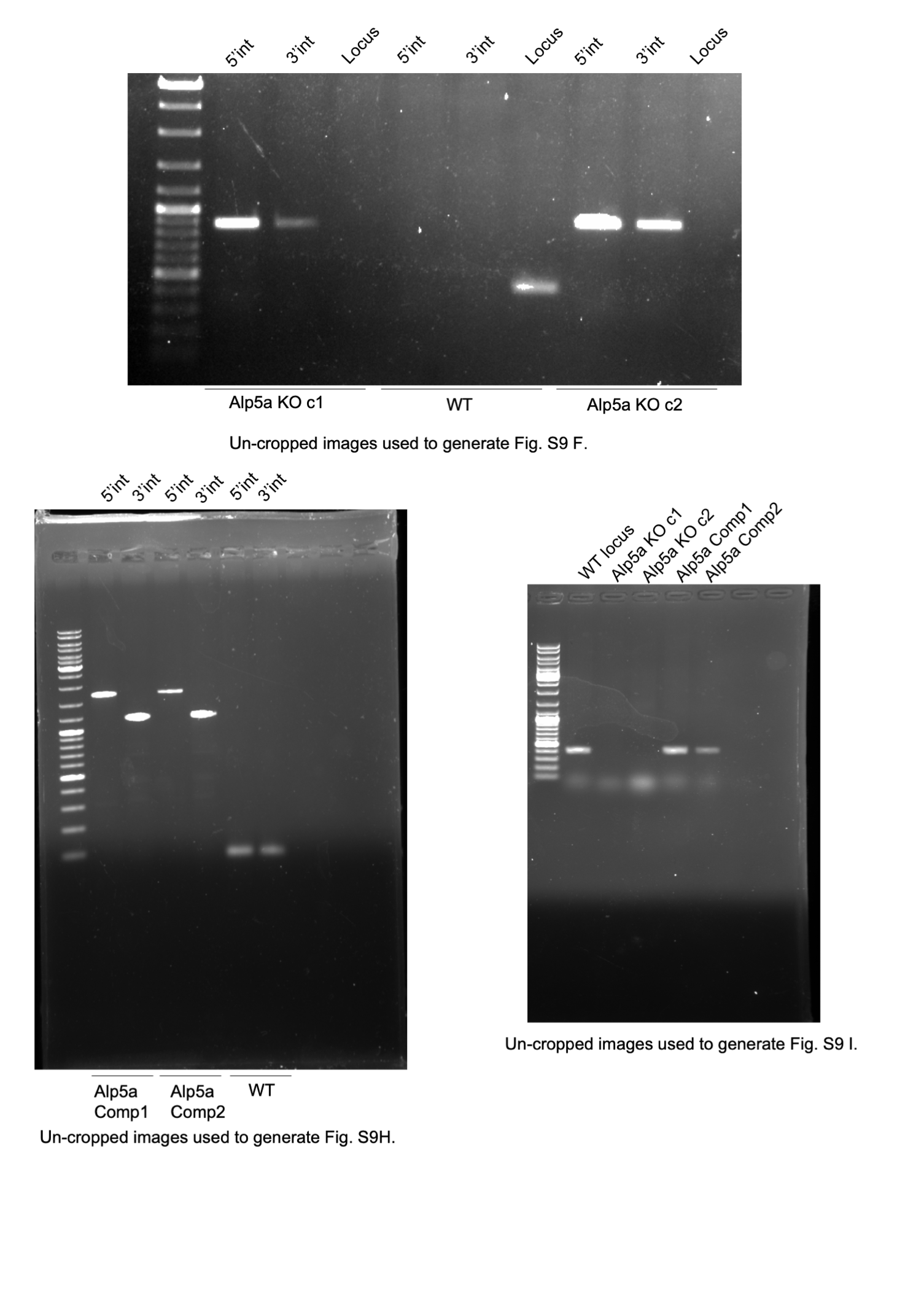


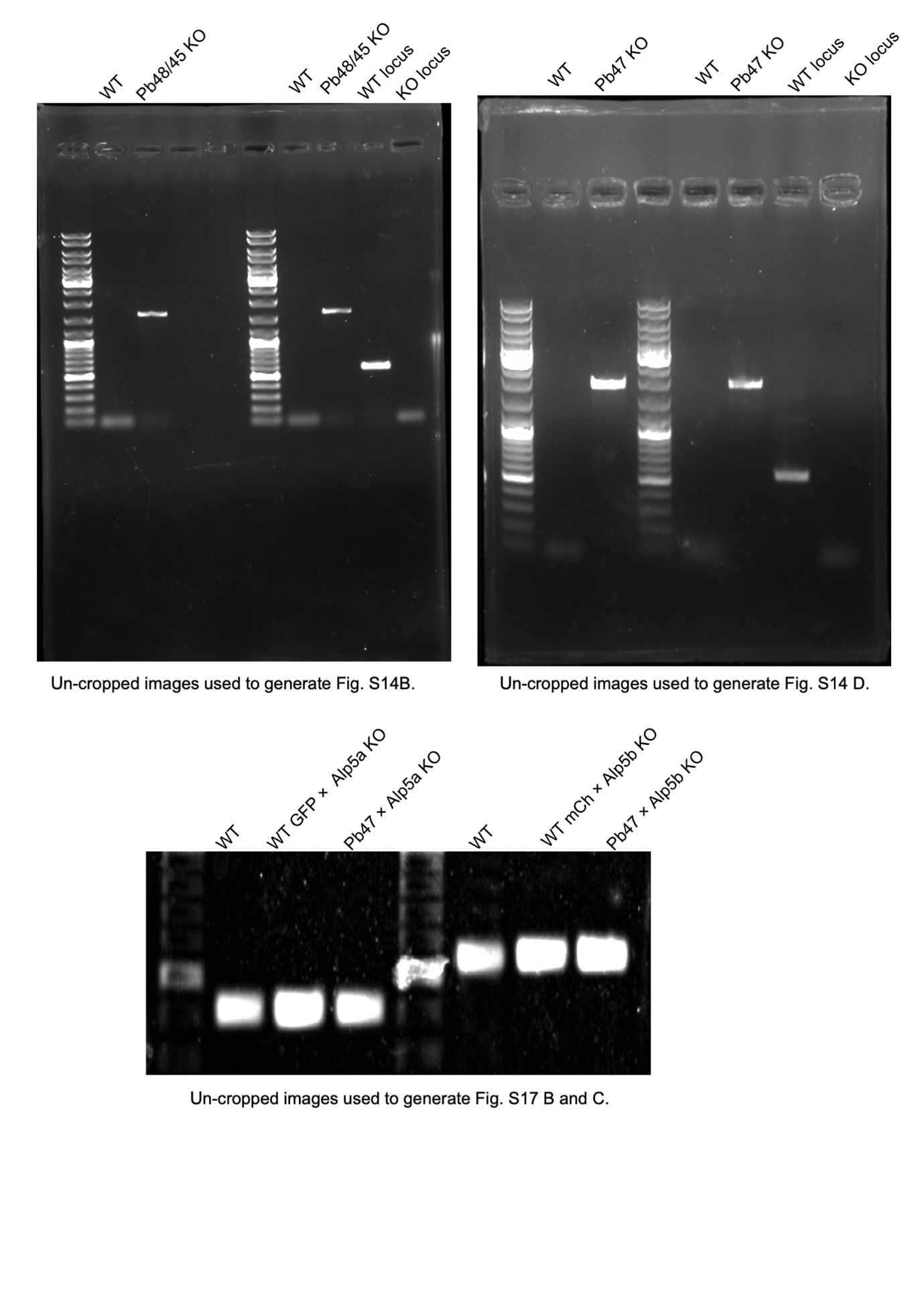

Supplement: S1 File — Uncropped images of all the western blots and gels used in main and supplementary figures. Blots and gels are labelled with respective figures and panels. (DOCX) [file ppat.1013687.s021.docx]
