# Supplementary material for: Plasmodium actin-like proteins are essential for DNA segregation during male gametogenesis and malaria transmission
Source: PLoS Pathog. 2025 Nov 11;21(11):e1013687. doi: 10.1371/journal.ppat.1013687 (PMC12617974; doi:10.1371/journal.ppat.1013687)
Supplement: S1 Table — (DOCX) [file ppat.1013687.s018.docx]

**S1 Table.** List of primers used in this study.

| **Primer** | **Primer sequences** | **Restriction site** |
| --- | --- | --- |
| 1611 | GTCGAC TCCTATTCTTATCAAATTTACCCTA | *Sal*I |
| 1612 | GTCGACACGGGAACATTTTTCCTGTCC | *Sal*I |
| 1613 | GCGGCCGCTGGGCAAACATCCTATAAAGG | *Not*I |
| 1614 | GGCGCGCCGTGCTAGAACAATTTGGATATAT | *Asc*I |
| 1665 | GATTATGCGGGTATTCATATTTGCATC | NA |
| 1666 | TAACTATGAAAGAAGGTGAAAATGAAAATAGTG | NA |
| 1671 | GAATCAAATAGTTATGTAAGTCTA | NA |
| 1672 | TAGGATGTTTGCCCAAATAAAAA | NA |
| 1675 | CTCGAGGAATCAAATAGTTATGTAAGTCTA | *Xho*I |
| 1676 | AGATCTTAGGATGTTTGCCCAAATAAAAA | *Bgl*II |
| 1677 | GCGGCCGCAGGTGTAGAGAACAAGAAAATA | *Not*I |
| 1724 | TTCCTGTGGCTGAAAATAATG | NA |
| 1895 | TATAGGGCGAATTGGGTACCATAATTCGATTGTGTATTTTTATT | *Kpn*I |
| 1896 | GTATATTTTCCATCGATGCTCATTTTATTTTACATTTATTA | *Cla*I |
| 1897 | TGCAAGCTTGCGGCCGCTAATTTTTTTTTTGCATATAAGAAG | *Not*I |
| 1898 | ATTACGCCAGGCGCGCCACTTTCATCTTATTATATTTGTTTC | *Asc*I |
| 2089 | GTCTATGCTTTTCTATCATGC | NA |
| 2090 | AGCAGGGGATGGTTATTTTA | NA |
| 2091 | CGGACTCATATAATAATTTGC | NA |
| 2092 | ATACAATAACTTTCCTTGTAAG | NA |
| 2128 | CGGGCCCCCCCTCGAGCTCCCCAAAATAAAGATGAATCTG | *Xho*I |
| 2129 | CGTATGGGTAAGATCTATACAATAACTTTCCTTGTAAGCTC | *Bgl*II |
| 2130 | CCGACTTAACTAGCATATATT | NA |
| 2268 | CAGTCGACGGTATCGATATAATTCGATTGTGTATTTTT | *Cla*I |
| 2269 | TGGGCTGCAGGAATTCATATAAATATATCACATACTTTCA | *EcoR*I |
| 2270 | GCTTGACCATGATTACGCC | NA |
| 2271 | TATTATAATTAATGTAAGCG | NA |
| 2272 | ATCAAGATAAATATACCATAAGT | NA |
| 2229 | TAATTTAGGTATGAAATGTAGG | NA |
| 2230 | GATGGAAGAAGTGCATTAGT | NA |
| 2231 | CTGGAATTATAGTACCAGGG | NA |
| 2232 | GTTCCCAAAAAAGTAGAGCA | NA |
| 2233 | CTGAAGACAGCGCACACA | NA |
| 2234 | GCCACCAACATTGCTAAAAA | NA |
| 2035 | CATACTAGCCATTTTATGTG | NA |
| 2036 | CTTTGGTGACAGATACTAC | NA |
| 1215 | GTTGTCTCTTCAATGATTCATAAATAG | NA |
| 1225 | TTCCGCAATTTGTTGTACATA | NA |
| 1392 | GCCCTCCATGTGCACCT | NA |
| 1218 | TACAACAAAAGGAGGTACAC | NA |
| 1913 | ACCAACTCAATTTAATAGATGT | NA |
