# Supplementary material for: Plasmodium actin-like proteins are essential for DNA segregation during male gametogenesis and malaria transmission
Source: PLoS Pathog. 2025 Nov 11;21(11):e1013687. doi: 10.1371/journal.ppat.1013687 (PMC12617974; doi:10.1371/journal.ppat.1013687)
Supplement: S2 Table — (DOCX) [file ppat.1013687.s019.docx]

**S2 Table.** **Protein-protein interaction prediction score.**

| **S.no.** | **Protein-protein interaction** | **Center** | **Lowest energy** |
| --- | --- | --- | --- |
|  | **AlphaFold Structure** | | |
| 1 | PbAlp5a-Alp5b | -636.9 | -773.4 |
| 2 | PbActin1-Alp5a | -1182.2 | -1187.1 |
| 3 | PbActin-Alp5b | -603.0 | -679.8 |
| 4 | PbActin2-Alp5a | -1258.2 | -1369.1 |
| 5 | PbActin 2-Alp5b | -680.8 | -844.8 |
|  | **Experimental high-resolution structures** | | |
| 1 | PfActin1-Alp5a | -1117.0 | -1166.2 |
| 2 | PfActin1-Alp5b | -705.4 | -831.0 |
| 3 | PbActin2-Alp5a | -786.2 | -1144.5 |
| 4 | PbActin2-Alp5b | -747.6 | -826.5 |
|  | **AlphaFold 3 prediction score** |  |  |
|  | **Protein-protein interaction** | **pTM** | **iPTM** |
| 1 | PbAlp5a-Alp5b | 0.45 | 0.15 |
| 2 | Arp2-Arp3 | 0.64 | 0.42 |
| 3 | PfActin1-Alp5a | 0.62 | 0.53 |
| 4 | PfActin1-Alp5b | 0.77 | 0.71 |
| 5 | PbActin2-Alp5a | 0.6 | 0.48 |
| 6 | PbActin2-Alp5b | 0.74 | 0.67 |
| 7 | Alp5a-Alp5b-F-actin | 0.4 | 0.19 |
| 8 | Arp2-Arp3-F-actin | 0.44 | 0.18 |
